# Agreeing a set of biopsychosocial variables for collection across the UK Eating Disorders Clinical Research Network: a consensus study using adapted nominal group technique

**DOI:** 10.1136/bmjment-2025-301760

**Published:** 2025-07-20

**Authors:** Tom Jewell, Iona Smith, James Downs, Anna Carnegie, Saakshi Kakar, Laura Meldrum, Lu Qi, Una Foye, Chelsea M Malouf, Suzanne Baker, Hope Virgo, Marilyn Okoro, Jessica Griffiths, Daniel Munblit, Moritz Herle, Ulrike Schmidt, Sarah Byford, Sabine Landau, Clare Llewellyn, Dasha Nicholls, Agnes Ayton, Sheryllin McNeil, Stephen Anderson, Gerome Breen, Karina L Allen

**Affiliations:** 1 Florence Nightingale Faculty of Nursing, Midwifery & Palliative Care, King’s College London, London, UK; 2 Psychological and Mental Health Services, Great Ormond Street Hospital for Children NHS Foundation Trust, London, UK; 3 Social Genetic & Developmental Psychiatry Centre, King’s College London, London, UK; 4 UK National Institute for Health and Social Care Research (NIHR) Biomedical Research Centre for Mental Health, South London and Maudsley NHS Foundation Trust, London, UK; 5 Lived Experience Steering Committee, Eating Disorders Clinical Research Network, King's College London, London, UK; 6 Department of Health Service and Population Research, King’s College London Institute of Psychiatry Psychology & Neuroscience, London, UK; 7 South London and Maudsley NHS Foundation Trust, London, UK; 8 Department of Paediatrics and Paediatric Infectious Diseases, I M Sechenov First Moscow State Medical University, Moscow, Russian Federation; 9 Centre for Eating and Weight Disorders, King’s College London Institute of Psychiatry Psychology & Neuroscience, London, UK; 10 Eating Disorders Outpatients Service, South London and Maudsley NHS Foundation Trust, London, UK; 11 King’s College London Clinical Trials Unit, London, UK; 12 Department of Biostatistics and Health Informatics, King’s College London Institute of Psychiatry Psychology & Neuroscience, London, UK; 13 Department of Epidemiology and Public Health, University College London, London, UK; 14 Department of Brain Sciences, Imperial College London, London, UK; 15 Cotswold House, Oxford, Oxford Health NHS Foundation Trust Adult Mental Health Services, Oxford, UK; 16 Birmingham Women’s and Children’s Hospitals NHS Foundation Trust, Birmingham, UK; 17 NHS Greater Glasgow and Clyde, Glasgow, UK

**Keywords:** Eating disorders

## Abstract

**Background:**

Eating disorders are serious psychiatric disorders associated with high levels of co-occurring physical and mental health conditions and poor treatment outcomes. The collection of standardised, routinely collected data within clinical services holds promise to improve patient care.

**Objective:**

To agree on a set of biopsychosocial variables for routine data collection within eating disorder services in the UK.

**Methods:**

Two online workshops were conducted using an adapted nominal group technique to agree on priorities for data collection in adult and child/adolescent eating disorder services. Workshop participants (n=43) consisted of people with lived experience, carers, clinicians and researchers. Two researchers independently conducted a reflexive thematic analysis of the workshop transcripts to identify qualitative priorities for data collection. Descriptive statistics were used to analyse the results of online voting.

**Findings:**

Thematic analysis identified four superordinate themes for data collection in eating disorder services: (1) a mutually valued and beneficial collaboration; (2) a holistic approach; (3) a balance between standardisation and individualisation; (4) doing no harm. Quantitative analysis of voting identified priorities across a range of domains, leading to a proposed biopsychosocial dataset.

**Conclusions:**

This project agreed on a set of biopsychosocial variables for routine data collection in the UK Eating Disorders Clinical Research Network. Further research should evaluate the implementation success of these variables.

**Clinical implications:**

Patients, caregivers and clinicians support routine data collection in eating disorder services so long as the measures used are considered meaningful, not overly burdensome, non-stigmatising and collected in collaboration between patients and treatment providers.

**WHAT IS ALREADY KNOWN ON THIS TOPIC**

- Eating disorder services do not collect the same routine data. This hampers efforts to understand who is being seen in services and what their clinical outcomes are.

**WHAT THIS STUDY ADDS**

- This study reports a consensus process to develop a set of biopsychosocial variables to be collected across a clinical research network in the United Kingdom.
- The agreed set of variables reflects shared priorities across lived experience, clinical and research perspectives.
- This clinical research network is the first to integrate biological and physiological variables into routine outcome collection in eating disorders.

**HOW THIS STUDY MIGHT AFFECT RESEARCH, PRACTICE OR POLICY**

- Routine collection of biopsychosocial variables across the network could facilitate the development of personalised treatment approaches in eating disorders.
- The study offers a practical model for collaborative dataset development and holds potential to influence national approaches to data collection, policy and service design in eating disorders.

## Introduction

 Eating disorders (EDs) are distressing and disabling disorders with high rates of co-occurring psychiatric and physical conditions.^1^ Available treatments are moderately effective: approximately 50% of patients who start treatment will complete it, and approximately 50% of patients who complete treatment will experience sustained symptom remission.^2^ Currently, there is a lack of standardisation in routine outcome collection for EDs,^3^ both nationally and internationally. This presents several barriers to progress in the field. First, routine outcome collection using harmonised measures across services can increase knowledge about the effectiveness of care, including specific treatments and service models. Second, when collected at scale, such data can inform the personalisation of treatment pathways, including precision medicine approaches for individual patients based on the analysis of large datasets.^4^ Finally, routine outcome collection at regular intervals combined with progress feedback, in which patients’ progress is actively monitored as part of therapy, can improve outcomes for patients within a treatment episode.^5^

To our knowledge, there have previously been two consensus exercises to establish standardised datasets in EDs, both of which used Delphi methodology, an expert consensus method. Bryant *et al*^6^ developed a proposed minimum dataset for Australia, while Austin *et al*^3^ conducted an international Delphi as part of the International Consortium on Health Outcome Measurement (ICHOM). Bryant *et al*^6^ recommended two ‘tiers’ of minimum data, with the ‘required’ tier comprising a relatively succinct list of variables including ED diagnosis, body mass index (BMI), comorbidities and treatment delivered, with a longer ‘optional’ tier including additional biological markers (eg, electrolytes) and treatment characteristics.

In contrast, Austin *et al*^3^ recommended specific outcome measures, following a systematic review and assessment of psychometric properties. Proposed measures covered ED symptomatology, depression/anxiety symptoms and quality of life. A range of demographic and clinical variables were also proposed, but only BMI, menstrual status and vital status were included as recommended biological variables. Given accumulating evidence of the influence of genetics on EDs^7^ and the promise of genomics and biomarker research to improve personalisation of treatment,^4 8^ the routine collection of biological variables such as endocrine markers could increase the opportunities for new insights.

The Eating Disorders Clinical Research Network (EDCRN) is a new initiative to create a United Kingdom (UK)-wide network of ED services,^9^ involving routine collection of data across biopsychosocial domains. Participating services will collect the same standardised information from patients, services and carers where applicable, using a secure digital platform. Individual patients and therapists will be able to review patient data, facilitating use for progress feedback and increasing the transparency and availability of data collected. A consensus exercise was chosen to inform variable selection for the EDCRN dataset drawing on the perspectives of people with lived experience (PWLE), carers, clinicians and researchers.

### Aims

This study aimed to agree on a set of standardised outcome variables to be implemented across UK ED services as part of the EDCRN project.

## Method

This study was conducted in line with the ACCORD reporting guideline for consensus methods^10^ (see online supplemental 1). The study was not preregistered.

### Steering committee

The consensus exercise was led by KLA, a Clinical Psychologist, and GB, a Professor of Psychiatric Genetics, both with expertise in ED research and joint Principal Investigators for the EDCRN. The steering committee included research and clinical collaborators (TJ, MH, US, SB, SL, CL, DN, AA, SM and SA) as well as lived experience collaborators (see below). AC, as EDCRN Study Coordinator, chaired the workshops with support from KLA and GB. Breakout rooms were led by GB, SK, TJ and UF.

### Patient and public involvement

A Lived Experience Steering Committee (JD, SB, HV, MO, JG) was formed prior to the start of the project and contributed to the consensus-building work presented in this study. Lived Experience Advisory Panels, consisting of separate youth, adult and carer panels, were established after the workshops took place, and were consulted at later stages of the process.

### Scoping

We searched for relevant literature in October 2023 and drew on two recent consensus exercises in the ED field.^3 6^ Given the recency of the international Delphi study, which included a systematic review of psychometric properties of relevant measures,^3^ we decided against conducting our own systematic review and Delphi to avoid duplication. We used adapted nominal group technique workshops^11^ to guide our final choice of variables to provide a more efficient methodology, thereby enabling faster implementation of the EDCRN in practice. We discussed the ICHOM outcome set^3^ with our Lived Experience Steering Committee, coinvestigators and clinical collaborators. We adapted the ICHOM set for this project to add biomarkers and reduced the number of demographic and treatment variables to aid implementation. Our choice of questions for the workshops was developed in collaboration with our Lived Experience Steering Committee and clinical and research collaborators and are presented in online supplemental file 2.

### Recruitment

Recruitment for the workshops occurred between December 2023 and February 2024. Information was shared on social media and via email lists aimed at PWLE and professional organisations associated with EDs, including the charity Beat, the British Eating Disorder Society and the Royal College of Psychiatry Eating Disorders Faculty. Specific reference was made to individuals with lived/living experience of an ED and their carers, researchers and clinicians; and to involvement in workshops focused on the information collected in children and young people (CYP) and adult ED services. In selecting PWLE and carers, we prioritised voices that are typically under-represented in ED research, if more expressions of interest were received than available places. We defined ‘underrepresented’ as individuals with ED diagnoses other than anorexia nervosa; males, transgender and non-binary individuals; and individuals from black and ethnic minority backgrounds. Efforts were also made to ensure representation from a range of the different geographical regions of the UK. Expressions of interest were reviewed by three members of the study team (KLA, TJ and AC). For the CYP workshop, PWLE were eligible if aged at least 13 years; those between 13 and 16 years required parent/guardian consent. PWLE over the age of 18 were still eligible to take part in the CYP workshop if they had prior experience of child/adolescent ED services. For additional safeguarding and accessibility, prior to attending the workshops, PWLE participants and caregivers had a remote one-to-one meeting (or alongside a caregiver for participants under the age of 16) with a member of the study team (AC). During this call, participants were provided with detailed information about participating in the workshops and the support mechanisms in place. Ahead of the sessions, all participants received a summary of variables identified in prior consensus-building studies, which formed the starting point for discussions in the workshops.^3 6^ Participants received a £20 shopping voucher for taking part.

### Adapted nominal group technique workshops

Two adapted nominal group technique^11^ workshops were conducted online to inform priorities for data collection within the EDCRN. Nominal group technique is a structured method for obtaining consensus in groups, originally comprised of four stages: (1) silent generation of ideas; (2) ‘round robin’ feedback; (3) clarification of ideas and (4) voting. We adapted the method to include a round of voting at the start of the workshop to orient participants to the range of variables being considered as well as to assess priorities prior to group discussions.

The first workshop, focussing on adult ED services, took place on 26 January 2024, and the second, on CYP ED services, was held on 16 February 2024. Facilitators of the workshops were members of the research team and our Lived Experience Steering Committee.

Workshops were open to participants from any of the following backgrounds: PWLE, carers, clinicians and researchers. We aimed to recruit roughly equal numbers of each group but received higher levels of interest from PWLE and carers. Piloting of workshop materials included testing of online voting and participation methods. Our adapted nominal group technique workshops used the following method:

1. **Introduction.** This included an explanation of the aims of the EDCRN project, and the purpose of the workshops.
2. **Round 1 of voting on priority variables**. Participants were asked to rate and rank the importance of including factors in the minimum dataset. Participants rated the importance of recording individual items on a scale from 0 (optional) to 10 (essential) for the following domains: ED factors (eg, previous and current ED diagnoses), treatment types (eg, inpatient, outpatient), routes to treatment (eg, admission and discharge date) and treatment received (eg, psychological therapy model/frequency). Participants were then asked to rank in order of priority the following domains: physical health factors; other mental health and neurodevelopment factors; demographics; and social factors. Voting was anonymously conducted using the Mentimeter online voting platform.^12^ Steering committee members and facilitators did not have voting rights.
3. **Small ‘breakout’ groups**. Breakout groups consisted of approximately 4–6 participants and two facilitators. Using the online platform Padlet,^13^ participants were asked to answer in writing, initially without verbal discussion, three questions: (1) which variables they considered most important; (2) any variables they thought were missing and (3) any proposed variables they disagreed on. Both adult and CYP workshops considered the same variables, comprising the following domains: ED factors, routes to treatment, treatment received, treatment type, demographic variables, other mental health and neurodevelopment, physical health and social factors. Written answers appeared as online ‘sticky notes’ on Padlet, which could be viewed by other participants.
4. **Small group discussion within ‘breakout’ groups**. Participants discussed each of the three questions from the Padlet in breakout groups in a facilitated discussion. To aid accessibility, participants could use the ‘chat’ function as an alternative to verbal contributions. To support adolescent participants to contribute, facilitators were careful to use plain English, to promote the use of the chat function and ensured that all small group members were invited to share their views.
5. **Feedback and discussion in the wider group**. A facilitator from each group presented key ideas from their breakout group discussion to the wider group. This was accompanied by an opportunity for further whole-group discussion.
6. **Round 2 of voting on priority variables**. Participants voted again using the Mentimeter platform with responses hidden during the voting process, answering the same questions as in Round 1, unless a variable had been dropped or revised. In the event of changes to variables, participants voted in Round 2 on the revised variable. Following this second round of voting, results of the votes were shared with the group and the workshops ended.

### Data synthesis and postworkshop survey

Following the workshops, the research team reviewed responses and compiled an initial draft of the dataset and timepoints for collection, informed by workshop participants and in concordance with prior consensus-building studies.^3 6^

Where there was consensus around domains, as opposed to specific measures, the research team typically adopted the applicable measure from the ICHOM dataset.^3^ Where reference was made to the value of specific outcome measures, these were included in the dataset. Conversely, where participants expressed concern with the utility of a particular measure, these were removed from the dataset.

Specialist clinical input was sought on physiological markers (eg, endocrine tests) and the feasibility of different blood tests, with 12 ED services sharing the blood tests routinely collected by their service.

After the above steps, the draft dataset was reviewed by members of EDCRN’s Lived Experience Steering Committee and EDCRN coinvestigators and clinical collaborators. In addition, the draft dataset was circulated to everyone who expressed interest in attending the workshop (including attendees) together with a short online survey. The survey asked whether there was anything on the list which respondents disagreed with and/or should be substituted for something else.

Following the survey, further suggestions were incorporated into the dataset. In the final phase, the updated dataset was shared again with workshop participants, EDCRN’s Lived Experience Steering Committee, the EDCRN Lived Experience and Carers and Supporters Advisory Boards and (clinical and academic) coinvestigators for final comment.

### Qualitative analysis

Workshops took place on Microsoft Teams and were recorded, transcribed and anonymised. Two researchers (JD and IS) verified and collated the transcripts before agreeing on a shared, stepwise approach to coding, aligned with Braun and Clarke’s six phases of reflexive thematic analysis: (1) data familiarisation, (2) generating initial codes, (3) constructing themes, (4) reviewing themes, (5) defining and naming themes and (6) presenting findings.^14^

During coding, the researchers generated initial codes that captured both semantic content and latent meanings within the data. All transcripts were double-coded. Reflexive journaling supported critical self-awareness, with researchers documenting and reflecting on their assumptions, positionality and decision-making throughout the process. In later stages, dialogue with ‘critical friends’^15^ (TJ, KLA) provided further analytic rigour.

### Quantitative analysis

Results of the two rounds of voting were reported using descriptive statistics, with the mean and SD calculated for all questions, which asked for prioritisation of variables on a 0–10 scale from optional (rated 0) to essential (rated 10). All variables with a mean score greater than 7 out of 10 were determined to be a priority. For questions asking for ranking of domains relative to each other, the top five options were determined as a priority.

## Results

1. Nominal group technique workshops

Discounting erroneous or fraudulent accounts (eg, ‘bots’, multiple submissions from the same IP address in a short space of time, or from outside the UK), we received 35 expressions of interest (EOI) from carers and 80 EOI from PWLE of EDs. We received fewer EOIs from clinicians and researchers, with all participants in these groups being invited to a workshop. Applications were reviewed by three members of the study team (KLA, TJ and AC).

The final sample attending the nominal group technique workshops totalled 43 participants, comprising 21 PWLE, 12 clinicians, 8 carers and 2 researchers. Demographic characteristics of the sample are presented in table 1. For the adult workshop, the mean age among PWLE was 34.4 years (SD=10.61, n=9), ranging between 19 and 49 years. For the child/adolescent workshop, the mean age of PWLE was 22 years (SD=5.95, n=12), ranging between 13 and 32 years. One PWLE participant in the adult-focused workshop and four participants in the child and adolescent-focused workshop did not disclose their age; all participants met the minimum age criterion of 13. Participants were asked to self-select which workshop they wished to attend (adult or CYP).

**Table 1:** Demographic characteristics of participants in the workshops

Demographics	Researchers		Clinicians		PWLE		Carers		Total
	n	%	n	%	n	%	n	%	%
n (%)	2	4.7	12	27.9	21	48.8	8	18.6	
Gender									
Male			1	8.3	3	14.3	2	25	14.0
Female	2	100	11	91.7	16	76.2	6	75	81.4
Non-binary					2	9.5			4.7
Location									
London	1	50	1	8.3	8	38.1	2	25.0	27.9
South-East England					3	14.3	3	37.5	14.0
South-West England	1	50	1	8.3			1	12.5	7.0
East of England			2	16.7			1	12.5	7.0
North-West England			1	8.3			1	12.5	4.7
North-East England					1	4.8			2.3
Yorkshire			1	8.3	1	4.8			4.7
East Midlands			4	33.3	1	4.8			11.6
Wales			2	16.7	2	9.5			9.3
Scotland					1	4.8			2.3
Undisclosed					4	19			9.3
Ethnicity									
Black	1	50			3	14.3			9.3
Mixed					2	9.5	1	12.5	7
White	1	50	12	100	12	57.1	7	87.5	74.4
Undisclosed					4	19			9.3
Eating disorder(s) experienced*									
AN					13	50	7	87.5	58.8
BN					2	7.7			5.9
BED					5	19.2			14.7
OSFED					1	3.8			2.9
ARFID					2	7.7			5.9
Other					3	11.5	1	12.5	11.8

* Several participants had experience of more than one eating disorder. Some of those in the researcher and/or clinician category also anecdotally shared that they had ED experience. This is not recorded in the figures above.

AN, anorexia nervosa; ARFID, avoidant/restrictive food intake disorder; BED, binge eating disorder; BN, bulimia nervosa; ED, eating disorder; OSFED, other specified feeding or eating disorder; PWLE, people with lived experience.

### Qualitative results

Four superordinate themes were identified, each comprising two inter-related subthemes. Superordinate themes, reflecting priorities for data collection in ED services, were: (1) a mutually valued and beneficial collaboration; (2) a holistic approach; (3) a balance between standardisation and individualisation; (4) doing no harm. The overall thematic structure, with illustrative quotes, is presented in table 2. Further examples of illustrative quotes for each subtheme are presented in online supplemental 3.

**Table 2:** Thematic structure

Themes	Subthemes	Illustrative quote/s
**A mutually valued and beneficial collaboration** Participants emphasised that data collected by eating disorder services need to be both meaningful and of benefit to patients and other stakeholders.	**Addresses the challenges of treatment provision** The need for data collected to speak to challenges in treatment provision, such as waiting times and treatment effectiveness.	*‘The other thing I think’s quite important is the waiting time for treatment, because in my experience it seems to be the longer the wait is, the more severe symptoms tend to be by the time they get seen.’* (PWLE 7, Adult Workshop)
	**Viewed as worthwhile for everyone** Data collected need to be perceived as meaningful and have the potential for positive impacts on patients, carers, providers, and the advancement of knowledge in the field.	*‘Just recording our outcomes so that you can get paid by your commissioners is of value, but if it also helps you as a professional to risk profile the people in your care, it means that we can be so much more effective in the interventions we then take with individuals.’* (Clinician 3, CYP Workshop)
**A holistic approach** The importance of considering the whole person, including physical, psychological and socio-cultural factors.	**Balances a range of measures** The need to consider various factors and data points in treatment, recognising that everyone’s journey and needs are unique.	*‘I had other factors of trauma, physical disability, autism, and you know, if they weren't dealt with in the correct way, it made the motivation for change for eating disorder recovery much more difficult until they were addressed in a holistic way and bringing all those strands together and piecing them all up.’* (PWLE 5, Adult Workshop)
	**Covers biopsychosocial domains and neurodivergence** The importance of addressing biological, psychological, and social factors, as well as considering neurodivergence, in the treatment process.	*‘Autism screening should be part of eating disorders and assessment. Personally, that’s my feeling.’* (PWLE 3, Adult Workshop) *‘It’s important to recognise how social factors contribute to eating disorders. For example, people who lack a support network are at higher risk of severe outcomes.’* (PWLE 8, Adult Workshop)
**A balance between standardisation and individualisation** The need to balance standardised data collection with the flexibility necessary to address individual differences in treatment.	**Creates standardised, comparable data** The importance of standardised data collection to improve the quality of care and research as it promotes consistency and comparability across different settings.	*‘When data collection isn't standardised, it not only affects the quality-of-care patients receive but also the quality of research that relies on that data.’* (Clinician 1, Adult Workshop)
	**Is flexible to include differences** The need for flexibility in treatment to accommodate individual differences and unique experiences.	*‘There’s so many routes to it. And what we were saying with the one size doesn’t fit all.’* (PWLE 5, CYP Workshop) *‘You have to adapt your data collection methods to fit the context, the culture, and the specific needs of the patient population you're working with. Otherwise, you risk collecting data that isn't relevant or accurate.’* (PWLE 2, Adult Workshop)
**Doing no harm** The importance of ensuring that treatment processes do not cause harm by overburdening or stigmatising patients.	**Is non-stigmatising and sensitive** The need to build trust and reduce stigma by remaining sensitive and respectful when providing care.	*‘Relationship building is key before jumping into questionnaires. It’s important to reduce shame and build trust, particularly for people who feel vulnerable.’* (PWLE 12, CYP Workshop)
	**Does not overburden** The importance of avoiding high levels of participant burden.	*‘How do we assess what’s most relevant without overburdening people with too many measures? We need a balance between gathering essential data and not overwhelming the patient.’* (Clinician 3, CYP Workshop)

CYP, children and young people; PWLE, person with lived experience.

### Quantitative results

Data used for quantitative analyses are available at https://osf.io/9chs3.^16^

### Rating of individual items as optional/essential

Two ED factors were reported as essential from the first round of the CYP workshop: ‘ED behaviours/cognitions’ and ‘current ED diagnosis’. In the second round, the group also determined ‘age of onset of ED’ and ‘previous ED diagnoses’ as essential. For the adult workshop, in the first round participants identified ‘current ED’, ‘ED behaviours/cognitions’, ‘previous ED diagnoses’, ‘previous ED treatment’ and ‘age of onset of ED’ as essential. In the second round, based on group discussion, a new factor, ‘impact on day-to-day life’ was added to the voting and determined to be an essential factor.

Considering routes to treatment, both the CYP and adult workshops determined ‘waiting time for treatment’ and ‘waiting time for assessment’ to be essential in the first round. In the second round, both the CYP and adult workshops added ‘admission and discharge date’. The adult workshop added a new variable, based on discussion, ‘distance from treatment’, which was rated as essential.

For treatment received, the essential factors at both workshops on the first round were ‘psychological therapy model/frequency’, ‘medication type/dose’, ‘dietetic support—type/frequency’ and ‘family/carer intervention’. In round 2, the essential factors for treatment received were unchanged for both workshops.

For treatment type, at round 1, both the CYP and adult workshop rated ‘referrals to other services during episodes of care’ (meaning referrals to non-ED services) as essential, with the adult workshop also rating the following as essential: ‘outpatient’, ‘inpatient’ and ‘voluntary/involuntary’. In the second round, the CYP workshop added ‘public vs private’. The adult workshop added two new variables which were rated as essential: ‘intensive day patient’ and ‘step-up/step-down’.

Findings for the rating of items as optional to essential are presented visually in online supplemental figures 1–8.

### Ranking items by priority for collection

The top-ranked demographics at round 1 for the CYP workshop were, in order of descending priority: (1) sex assigned at birth; (2) gender identity; (3) ethnicity; (4) race and (5) relationship status. At round 2, priorities were: (1) sex assigned at birth; (2) ethnicity; (3) race; (4) gender identity and (5) work/postsecondary education status. The top-ranked demographics in the adult workshop at round 1 were: (1) sex assigned at birth; (2) gender identity; (3) ethnicity; (4) race and (5) sexual orientation. In round 2, age was added as a variable and the order was: (1) age; (2) sex assigned at birth; (3) gender identity; (4) ethnicity and (5) sexual orientation.

For other mental health and neurodevelopmental factors, the top five priorities for the CYP workshop at round 1 were: (1) suicidality; (2) depression; (3) anxiety disorders; (4) non-suicidal self-harm and (5) autism. In the second round of the workshop, suicidality remained the top-ranked other mental health factor, while triggering life events was introduced as the second highest-ranked other mental health factor, followed by depression, anxiety disorders and autism. For the adult workshop, in the first round, the top five priorities were: (1) suicidality; (2) autism; (3) non-suicidal self-harm; (4) depression and (5) PTSD and complex-PTSD (c-PTSD). Suicidality and autism remained in first and second place in the second round, while PTSD and c-PTSD were ranked third, followed by depression and anxiety disorders.

For physical health factors, in the CYP workshop, the top five priorities in round 1 were: (1) cardiac health; (2) cognitive function; (3) weight change in recent months; (4) endocrine (hormonal) function and (5) electrolytes. At round 2, endocrine (hormonal) function was ranked slightly above weight change in recent months. For the adult workshop, in the first round, the top five priorities were: (1) cardiac health; (2) electrolytes; (3) weight change in recent months; (4) cognitive function and (5) formal diagnosis of malnutrition. In round 2, the only change was that cognitive function ranked above weight change.

The social factors which were ranked as the highest priority in the first round of the CYP workshop were: (1) marginalisation; (2) living arrangements; (3) financial stress; (4) housing security and (5) alcohol and tobacco consumption. In round 2, a new factor, external influences (eg, close friends or social media), was introduced as the third highest ranked factor, with financial stress ranked fifth. For adults, the highest priority in the first round were: (1) financial stress; (2) housing security; (3) marginalisation; (4) living arrangements and (5) alcohol and tobacco consumption. In round 2, caring responsibilities were added and voted as the top priority, followed by financial stress, marginalisation, housing security and living arrangements.

Findings for ranked items are presented visually in online supplemental figures 9–16.

1. **Data synthesis and postworkshop survey**

Following the workshop, the Autism Quotient 10^17^ was added to the EDCRN minimum dataset following high ratings for the importance of including autism. Motivation to change, a proposed variable in the ICHOM outcome set,^3^ was removed from EDCRN as it was seen as potentially harmful if it led to treatment being withdrawn due to ‘low motivation to change’. In some cases, there was consensus about the importance of assessing a particular domain but no existing consensus on the most appropriate measure/s to use (eg, for assessing trauma, weight stigma, food insecurity). In these cases, further consultation was conducted to seek lived experience, clinical and academic input on possible assessment tools, alongside a review of available measures and their psychometric properties. It was not possible to reach a consensus on an appropriate measure of therapeutic alliance (identified as a positive alternative to measures of motivation to change) and as such, a single question on ‘treatment experience’ was proposed for inclusion. For ED behaviours/cognitions, several measures were chosen to ensure coverage across ED diagnoses, including binge eating disorder and avoidant/restrictive food intake disorder, with weekly measurement included in line with progress feedback principles.^5^ It was decided that the default setting should be for EDCRN participants to be presented with all measures in the ED behaviours/cognitions category, but to reduce burden and aid personalisation, clinicians can ‘turn off’ measures if inappropriate, or they can be skipped by EDCRN participants.

Views of workshop participants also informed the demographic and treatment information included in the dataset, for example, information on waiting times, distance travelled for treatment and physical health comorbidities. Discussions around personalisation and reducing patient burden informed the consideration of optional measures (to be selected by patients and/or clinicians) and the decision not to mandate the completion of any questionnaires or items.

Twenty-two participants completed the postworkshop survey. Of these, 17 identified as having lived experience, eight as caregivers, three as clinicians, two as researchers, with some respondents identifying with more than one group. The dataset was further refined based on this feedback, resulting in the addition of the Difficulties in Emotional Regulation Scale^18^ and information on family history of EDs and other mental health comorbidities being added.

After the survey, a proposed final dataset was shared with participants and EDCRN lived experience and clinical advisory boards for final feedback, leading to the addition of the Adult ADHD Self-Report Scale^19^ as an optional measure. Small revisions were made to the planned cadence for data collection in conversation with these groups.

### Final agreed measures and variables

The final list of agreed essential measures, biological/physiological markers and other variables to be collected by the EDCRN, including demographic variables, are presented in tables3  4. Optional self-report measures are presented in online supplemental 4, demographic variables reported by patients and caregivers in online supplemental file 5 and 6 respectively, and clinician and service-reported variables in online supplemental 7. In January 2025, the EDCRN launched in ED services. Services participating in the network have agreed to collect the dataset from patients receiving treatment under their care and their caregivers, facilitating the availability of large-scale standardised EDs data to enhance outcomes and improve patient care.

**Table 3:** Final agreed essential self-report measures

Variable	Timepoint(s)	Reporter	Tool
ED behaviours/cognitions	Assessment, start of regular treatment (if>1 month later than assessment), 3 monthly while in treatment, transition, if applicable (from IP to DP, etc.), discharge. Optional follow-up, if patient consents to being contacted after discharge	Patient and parent/guardian on behalf of child (Child)	NIAS (adult 18+) and BEDS-7 (adult 18+) or PARDI-AR-Q (child – 14–17) and parent (8–13)
ED behaviours/cognitions (cont.)	Assessment, start of regular treatment (if>1 month later than assessment), monthly while in treatment, transition, if applicable (from IP to DP, etc.), discharge, optional follow-up, if patient consents to being contacted after discharge	Patient and parent/guardian on behalf of child (Child)	EDE-Q (adult 18+) or EDE-A (13–17 years) or ChEDE-Q8 (child 8–12 years)
ED behaviours/cognitions—weekly measure	Weekly from start of regular treatment* *Ask alternative weeks to EDE-Q/A/ChEDE	Patient	ED-15 (adult 18+) ED-15-Y (child 8–17 years)
Depression	Assessment, start of regular treatment (if>1 month later than assessment), monthly while in treatment, transition, if applicable (from IP to DP, etc.), discharge optional follow-up, if patient consents to being contacted after discharge	Patient	PHQ-9 (adult 18+) RCADS-25 (child 8–17 years)
Anxiety	Assessment start of regular treatment (if>1 month later than assessment) monthly while in treatment transition, if applicable (from IP to DP etc.) discharge, optional follow-up, if patient consents to being contacted after discharge	Patient	GAD-7 (adult 18+) RCADS-25 (child 8–17 years)
Autism	Assessment, start of regular treatment (if>1 month later than assessment), 3 monthly while in treatment, transition, if applicable (from IP to DP, etc.), discharge, optional follow-up, if patient consents to being contacted after discharge	Patient	AQ-10 (adult/child age 12+)
OCD	Assessment, start of regular treatment (if>1 month later than assessment), 3 monthly while in treatment, transition, if applicable (from IP to DP, etc.), discharge, optional follow-up, if patient consents to being contacted after discharge	Patient	FOCI (adult 18+)—25-item C-FOCI (child 11–17 years) Or: OCI-R (adult 18+)—18 item OCI-CV-R (child 8–17 years)—18-item
Perfectionism	Assessment, start of regular treatment (if>1 month later than assessment), 3 monthly while in treatment, transition, if applicable (from IP to DP, etc.), discharge, optional follow-up, if patient consents to being contacted after discharge	Patient	CPQ (adult age 14+) CAPS (child 8–13)
Emotional regulation	Assessment, discharge	Patient	PID-5-BF (adults 18+) DERS (Adolescent 13–17 years)
Psychosocial impairment	Assessment, start of regular treatment (if>1 month later than assessment), 3 monthly while in treatment, transition, if applicable (from IP to DP, etc.), discharge	Patient	CIA (adult 18+) and WSAS (adult 18+)—adapted to include study and work KIDSCREEN-10 (child 8–17 years)
Adverse childhood experiences	Assessment	Patient and parent/guardian on behalf of child (Child)	ACE-Q (adult 18+) PEARLS (child 12–17 years) or Parent/guardian (8–11)p
Self-injurious behaviour	Assessment, admission, transition (from IP to DP etc.), discharge, optional follow-up, if patient consents to being contacted after discharge	Patient	TAF_52_Worth (adult 18+—not validated)
Food insecurity	Assessment	Patient (adult) parent/guardian	Radimir/Cornell instrument (18+) or parent/guardian (8—17)

ACE-Q, Adverse Childhood Experiences Questionnaire; ADHD, attention-deficit/hyperactivity disorder; AQ-10, Autism Spectrum Quotient-10; BEDS-7, Binge Eating Disorder Screener-7; CAPS, Clinician-Administered PTSD Scale; C-FOCI, Children’s Florida Obsessive-Compulsive Inventory; ChEDE-Q8, Child Eating Disorder Examination Questionnaire; CIA, Clinical Impairment Assessment Questionnaire; CPQ, Child Perceptions Questionnaire; DERS, Difficulties in Emotion Regulation Scale; DP, day patient; ED-15, Eating Disorder-15; ED, eating disorder; EDE-A, Eating Disorder Examination—Adolescent; EDE-Q, Eating Disorder Examination Questionnaire; ED-15-Y, Eating Disorder—15 for Youth; FOCI, Florida Obsessive-Compulsive Inventory; GAD-7, Generalised Anxiety Disorder-7; IP, inpatient; KIDSCREEN-10, KIDSCREEN-10 Index; NIAS, Nine Item ARFID Screen; OCD, obsessive compulsive disorder; OCI-CV-R, Obsessive-Compulsive Inventory—Children’s Version—Revised; OCI-R, Obsessional Compulsive Inventory—Revised; PARDI-AR-Q, Pica, ARFID and Rumination Disorder Interview ARFID Questionnaire; PEARLS, Paediatric ACEs and Related Life Events Screener; PHQ, Patient Health Questionnaire; PID-5-BF, Personality Inventory for DSM-5—Brief Form; PTSD, post-traumatic stress disorder; RCADS-25, Revised Children’s Anxiety and Depression Scale-25; TAF, thoughts and feelings.; WSAS, Work and Social Adjustment Scale.

**Table 4:** Final agreed biological/physiological variables

Variable	Timepoint(s)	Reporter	Test(s)
Weight history	Assessment	Patient	Highest and lowest weight as adult (via additional question on EDE-Q)
BMI (adults) % median BMI (children and adolescents)	Assessment, start of regular treatment (if>1 month later than assessment), 3 monthly while in treatment, transition, if applicable (from IP to DP, etc.), discharge	Clinician	Weight and height
Muscle power	Assessment, discharge	Clinician	Sit Up Squat Stand test (SUSS), Hand Grip Strength test (HGS)
Endocrine	Assessment, admission, discharge	Clinician from blood test results (where available)	IGF-1, cortisol, E2, testosterone, prolactin, thyroid function: LH, FSH, TSH, FT3, FT4, PTH, HbA1c, glucose, leptin.
Liver function	Assessment, admission, discharge	Clinician from blood test results (where available)	ALT, AST, ALP, albumin, bilirubin
Kidney function	Assessment, admission, discharge	Clinician from blood test results (where available)	Urea
Cardiac function	Assessment, admission, discharge	Clinician from blood test results (where available)	Full blood count, cardiac enzyme, BNP, CK
Electrolytes	Assessment, admission, discharge	Clinician from blood test results (where available)	Potassium, sodium, calcium, phosphate
Bone health	Assessment, admission, discharge	Clinician from blood test results (where available)	Bone profile
Other blood test markers	Assessment, admission, discharge	Clinician from blood test results (where available)	Vitamin D, iron, ferritin

ALP, Alkaline Phosphatase; ALT, Alanine Aminotransferase; AST, Aspartate Aminotransferase; BMI, body mass index; BNP, B-type Natriuretic Peptide; CK, Creatine Kinase; DP, day patient; EDE-Q, Eating Disorder Examination Questionnaire; FSH, Follicle-Stimulating Hormone; FT3, Free Triiodothyronine; FT4, Free Thyroxine; HbA1c, Glycated Haemoglobin; IGF-1, Insulin-like growth factor 1; IP, inpatient; LH, Luteinising Hormone; PTH, Parathyroid Hormone; TSH, Thyroid-Stimulating Hormone.

## Discussion

This study presents the findings of two adapted nominal group technique workshops, which were used to inform the development of a routinely collected outcome set with broad coverage across biopsychosocial domains. This builds on prior work in the field to gain consensus on variables for collection in ED services^3 6^ but is unique in adding a suite of biological/physiological markers. Our qualitative analysis provides insights into outcome set development and implementation of potential relevance beyond ED services, with support for routine data collection so long as data are meaningful, likely to help improve care or advance knowledge and implemented sensitively.

Strengths of our study include the integration of physiological/biological variables within a routinely collected ED outcome set. The use of workshops followed by further rounds of consultation with workshop participants and stakeholders, including our Lived Experience Steering Committee, clinical partners and researchers, allowed for the final EDCRN dataset to be informed by the priorities of all stakeholders, and adapted specifically to the context of UK services. However, the study has some weaknesses. Participants in our workshops with lived experience were predominantly White, from the South-East of England and with experience of anorexia nervosa. Additionally, despite efforts to proactively recruit this demographic, children and adolescents comprised a small number of participants in the CYP workshop. Voting was conducted anonymously, which meant that results could not be separated for different stakeholder groups. Readers should note that PWLE were the largest group across workshops. Our workshops were attended by relatively few researchers or clinicians, although their views were incorporated through processes outside of the workshops.

Future research is needed to investigate priorities for outcomes across a wider range of ED patients and carers, including people from ethnic minorities, and those with non-restrictive ED presentations, such as binge eating disorder. Qualitative studies employing purposive sampling of under-represented groups, as well as survey designs to achieve larger samples of participants, are both needed. Research into the implementation of the EDCRN into clinical settings is needed to understand the barriers and facilitators to its uptake.

## Supplementary material

10.1136/bmjment-2025-301760 online supplemental file 1

## Data Availability

Data are available in a public, open access repository.
