# Supplementary material for: Agreeing a set of biopsychosocial variables for collection across the UK Eating Disorders Clinical Research Network: a consensus study using adapted nominal group technique
Source: BMJ Ment Health. 2025 Jul 20;28(1):e301760. doi: 10.1136/bmjment-2025-301760 (PMC12278168; doi:10.1136/bmjment-2025-301760)
Supplement: online supplemental file 1 [file bmjment-28-1-s001.docx]

**Supplementary Materials for: Agreeing a set of biopsychosocial variables for collection across the UK Eating Disorders Clinical Research Network (EDCRN): A consensus study using adapted nominal group technique**

**Contents**

[Supplementary 1: ACCORD Checklist 2](#_Toc201331996)

[Supplementary 2: Figures 1-16 5](#_Toc201331997)

[Supplementary 3: Thematic structure with additional illustrative quotes 21](#_Toc201331998)

[Supplementary 4: Optional self-report measures 35](#_Toc201331999)

[Supplementary 5: Demographics and ED history Questionnaire – patients 36](#_Toc201332000)

[Supplementary 6: Demographics and ED history Questionnaire – caregivers 40](#_Toc201332001)

[Supplementary 7: Clinician and service reported variables 44](#_Toc201332002)

| **Supplementary 1: ACCORD Checklist** | | | |
| --- | --- | --- | --- |
| Item No. | Section | Checklist Item (*help text*) | Page No. |
| T1 | **Title** | Identify the article as reporting a consensus exercise and state the consensus methods used in the title.  *For example, Delphi or nominal group technique.* | 1 |
| I1 | **Introduction** | Explain why a consensus exercise was chosen over other approaches. | 3 |
| I2 |  | State the aim of the consensus exercise, including its intended audience and geographical scope (national, regional, global). | 3 |
| I3 |  | If the consensus exercise is an update of an existing document, state why an update is needed, and provide the citation for the original document. | N/a |
| M1 | **Methods**  Registration | If the study or study protocol was prospectively registered, state the registration platform and provide a link. If the exercise was not registered, this should be stated.  *Recommended to include the date of registration.* | 3 |
| M2 | Selection of SC and/or panellists | Describe the role(s) and areas of expertise or experience of those directing the consensus exercise.  *For example, whether the project was led by a chair, co-chairs or a steering committee, and, if so, how they were chosen. List their names if appropriate, and whether there were any subgroups for individual steps in the process.* | 3-4 |
| M3 |  | Explain the criteria for panellist inclusion and the rationale for panellist numbers. State who was responsible for panellist selection. | N/a. |
| M4 |  | Describe the recruitment process (how panellists were invited to participate).  *Include communication/advertisement method(s) and locations, numbers of invitations sent, and whether there was centralised oversight of invitations or if panellists were asked/allowed to suggest other members of the panel.* | 4-5 |
| M5 |  | Describe the role of any members of the public, patients or carers in the different steps of the study. | 4-5 |
| M6 | Preparatory research | Describe how information was obtained prior to generating items or other materials used during the consensus exercise.  *This might include a literature review, interviews, surveys, or another process.* | 4 |
| M7 |  | Describe any systematic literature search in detail, including the search strategy and dates of search or the citation if published already.  *Provide the details suggested by the reporting guideline PRISMA and the related PRISMA-Search extension.* | N/a. |
| M8 |  | Describe how any existing scientific evidence was summarised and if this evidence was provided to the panellists. | 5 |
| M9 | Assessing consensus | Describe the methods used and steps taken to gather panellist input and reach consensus (for example, Delphi, RAND-UCLA, nominal group technique).  *If modifications were made to the method in its original form, provide a detailed explanation of how the method was adjusted and why this was necessary for the purpose of your consensus-based study.* | 5-7 |
| M10 |  | Describe how each question or statement was presented and the response options. State whether panellists were able to or required to explain their responses, and whether they could propose new items.  *Where possible, present the questionnaire or list of statements as supplementary material.* | S2 |
| M11 |  | State the objective of each consensus step.  *A step could be a consensus meeting, a discussion or interview session, or a Delphi round.* | 5-7 |
| M12 |  | State the definition of consensus (for example, number, percentage, or categorical rating, such as ‘agree’ or ‘strongly agree’) and explain the rationale for that definition. | 8 |
| M13 |  | State whether items that met the prespecified definition of consensus were included in any subsequent voting rounds. | N/a. |
| M14 |  | For each step, describe how responses were collected, and whether responses were collected in a group setting or individually. | 6 |
| M15 |  | Describe how responses were processed and/or synthesised.  *Include qualitative analyses of free-text responses (for example, thematic, content or cluster analysis) and/or quantitative analytical methods, if used.* | 7 |
| M16 |  | Describe any piloting of the study materials and/or survey instruments.  *Include how many individuals piloted the study materials, the rationale for the selection of those individuals, any changes made as a result and whether their responses were used in the calculation of the final consensus. If no pilot was conducted, this should be stated.* | 5-6 |
| M17 |  | If applicable, describe how feedback was provided to panellists at the end of each consensus step or meeting.  *State whether feedback was quantitative (for example, approval rates per topic/item) and/or qualitative (for example, comments, or lists of approved items), and whether it was anonymised.* | 6-7 |
| M18 |  | State whether anonymity was planned in the study design. Explain where and to whom it was applied and what methods were used to guarantee anonymity. | 6 |
| M19 |  | State if the steering committee was involved in the decisions made by the consensus panel.  *For example, whether the steering committee or those managing consensus also had voting rights.* | 6 |
| M20 | Participation | Describe any incentives used to encourage responses or participation in the consensus process.  *For example, were invitations to participate reiterated, or were participants reimbursed for their time.* | 5 |
| M21 |  | Describe any adaptations to make the surveys/meetings more accessible.  *For example, the languages in which the surveys/meetings were conducted and whether translations or plain language summaries were available*. | 6-7 |
| R1 | Results | State when the consensus exercise was conducted. List the date of initiation and the time taken to complete each consensus step, analysis, and any extensions or delays in the analysis. | 5 |
| R2 |  | Explain any deviations from the study protocol, and why these were necessary.  *For example, addition of panel members during the exercise, number of consensus steps, stopping criteria; report the step(s) in which this occurred.* | N/a. |
| R3 |  | For each step, report quantitative (number of panellists, response rate) and qualitative (relevant socio-demographics) data to describe the participating panellists. | 9 |
| R4 |  | Report the final outcome of the consensus process as qualitative (for example, aggregated themes from comments) and/or quantitative (for example, summary statistics, score means, medians and/or ranges) data. | 11-17 |
| R5 |  | List any items or topics that were modified or removed during the consensus process. Include why and when in the process they were modified or removed. | 16 |
| D1 | Discussion | Discuss the methodological strengths and limitations of the consensus exercise.  *Include factors that may have impacted the decisions (for example, response rates, representativeness of the panel, potential for feedback during consensus to bias responses, potential impact of any non-anonymised interactions).* | 22 |
| D2 |  | Discuss whether the recommendations are consistent with any pre-existing literature and, if not, propose reasons why this process may have arrived at alternative conclusions. | 22 |
| O1 | Other information | List any endorsing organisations involved and their role. | N/a. |
| O2 |  | State any potential conflicts of interests, including among those directing the consensus study and panellists. Describe how conflicts of interest were managed. | 8 |
| O3 |  | State any funding received and the role of the funder.  *Specify, for example, any funder involvement in the study concept/design, participation in the steering committee, conducting the consensus process, funding of any medical writing support. This could be disclosed in the methods or in the relevant transparency section of the manuscript. Where a funder did not play a role in the process or influence the decisions reached, this should be specified.* | 8 |

# **Supplementary 2: Figures 1-16**


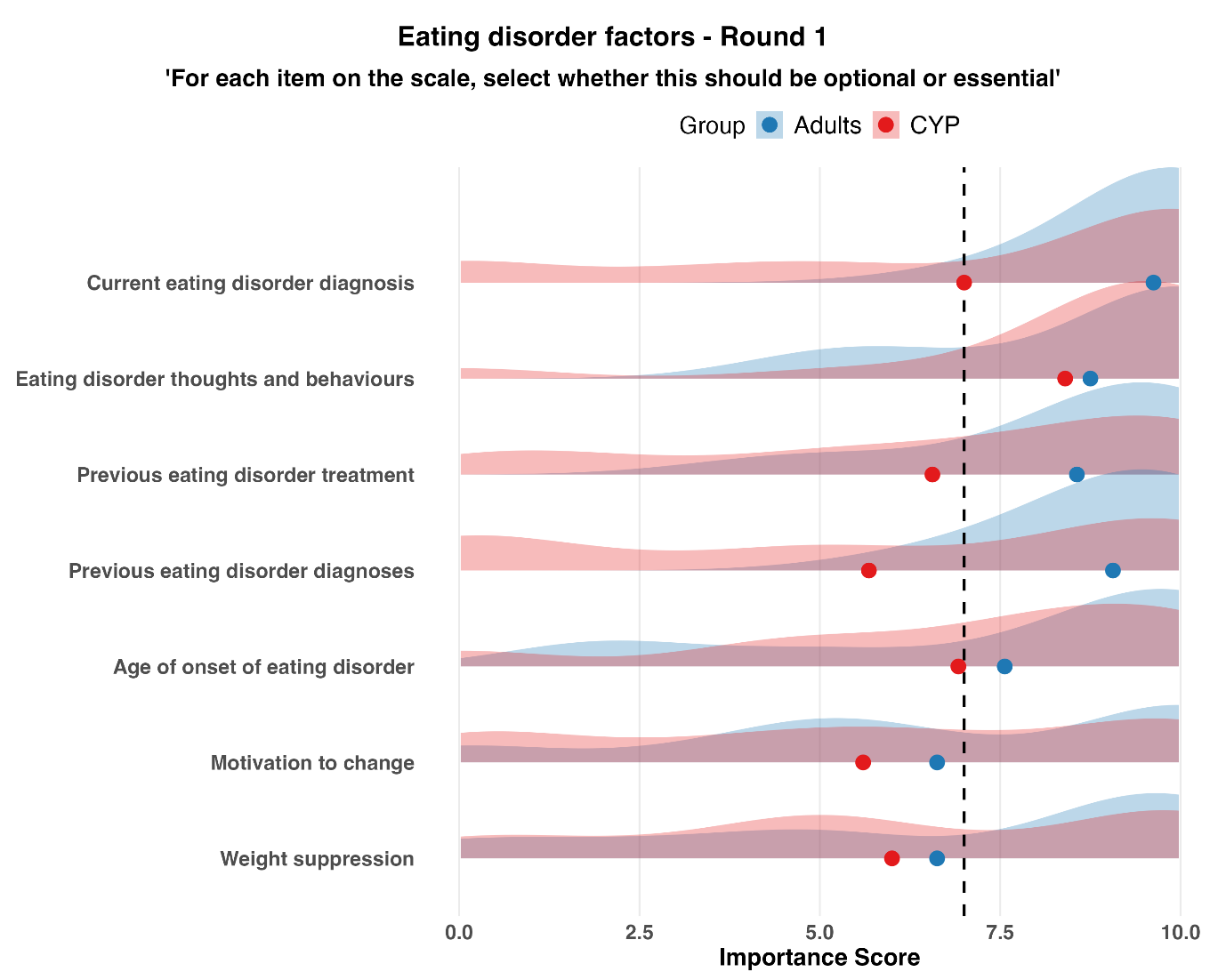


**Supplementary Figure 1: Voting on eating disorder factors at Round 1 for Adult and Children and Young People workshops.**

Notes: CYP = Children and Young People.


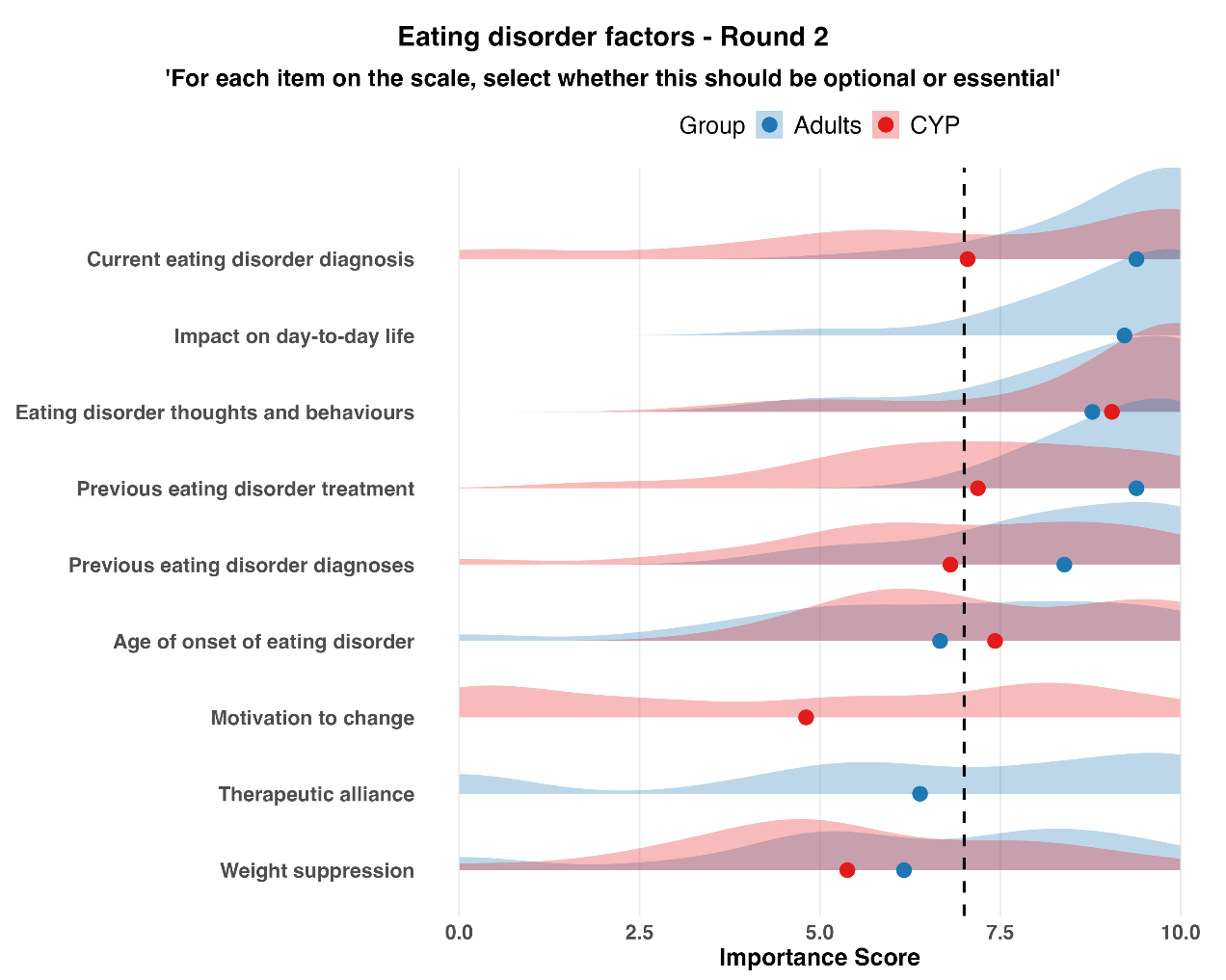


**Supplementary Figure 2: Voting on eating disorder factors at Round 1 for Adult and Children/Young People workshops.**

Notes: CYP = Children and Young People.

**
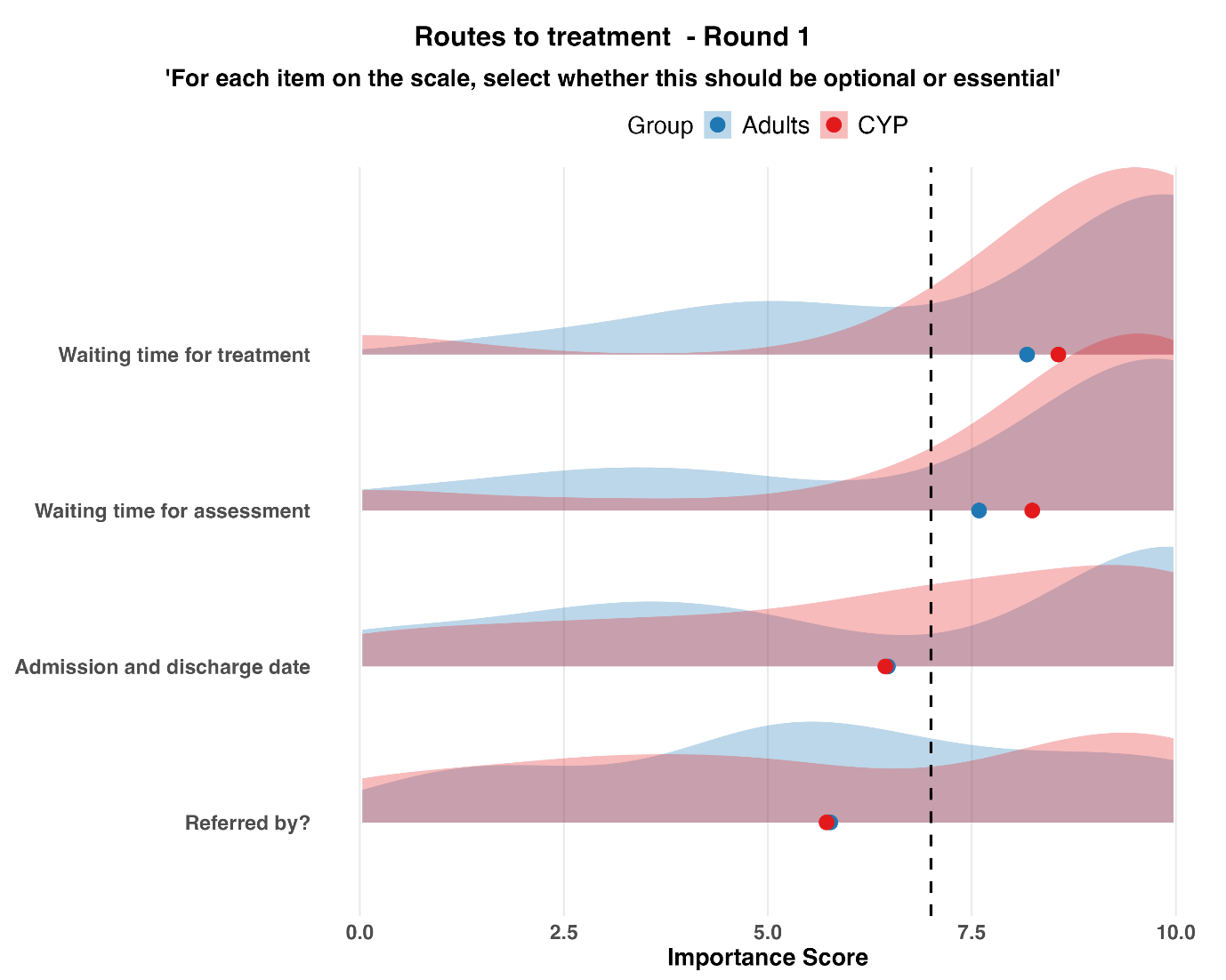
**

**Supplementary Figure 3: Voting on routes to treatment at Round 1 for Adult and Children/Young People workshops.**

Notes: CYP = Children and Young People.


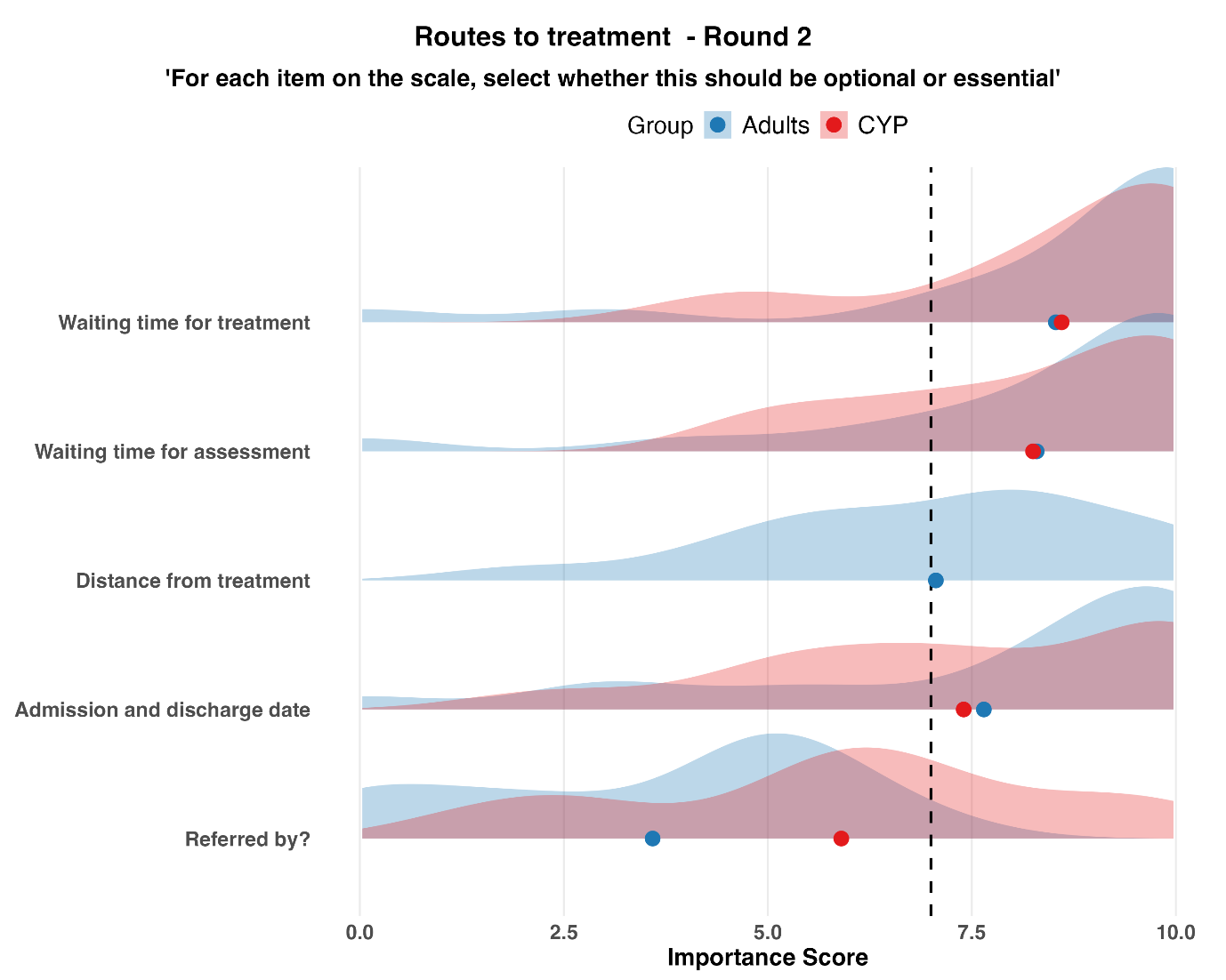


**Supplementary Figure 4: Voting on routes to treatment at Round 2 for Adult and Children/Young People workshops.**

Notes: CYP = Children and Young People.


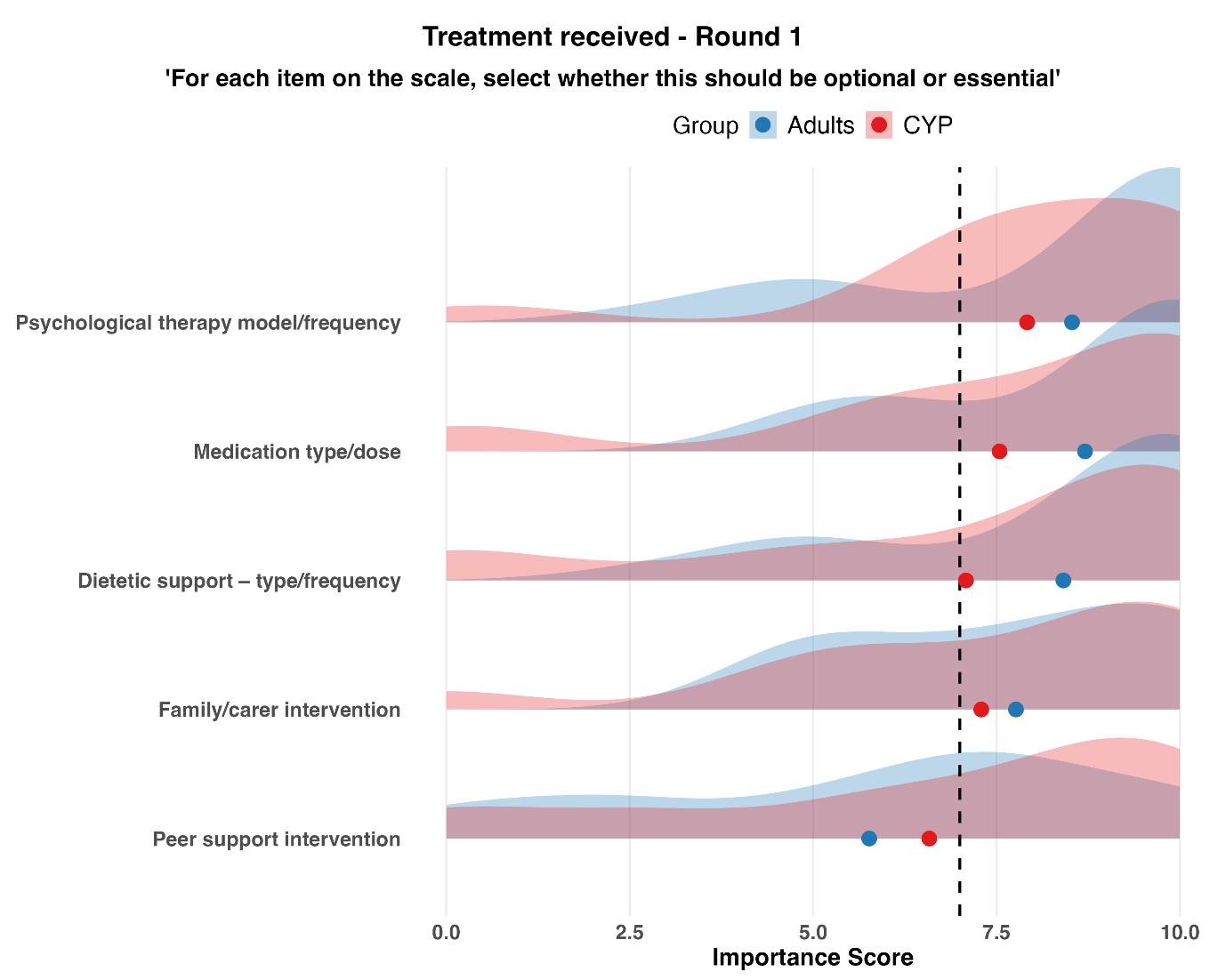


**Supplementary Figure 5: Voting on treatment received at Round 1for Adult and Children/Young People workshops.**

Notes: CYP = Children and Young People.


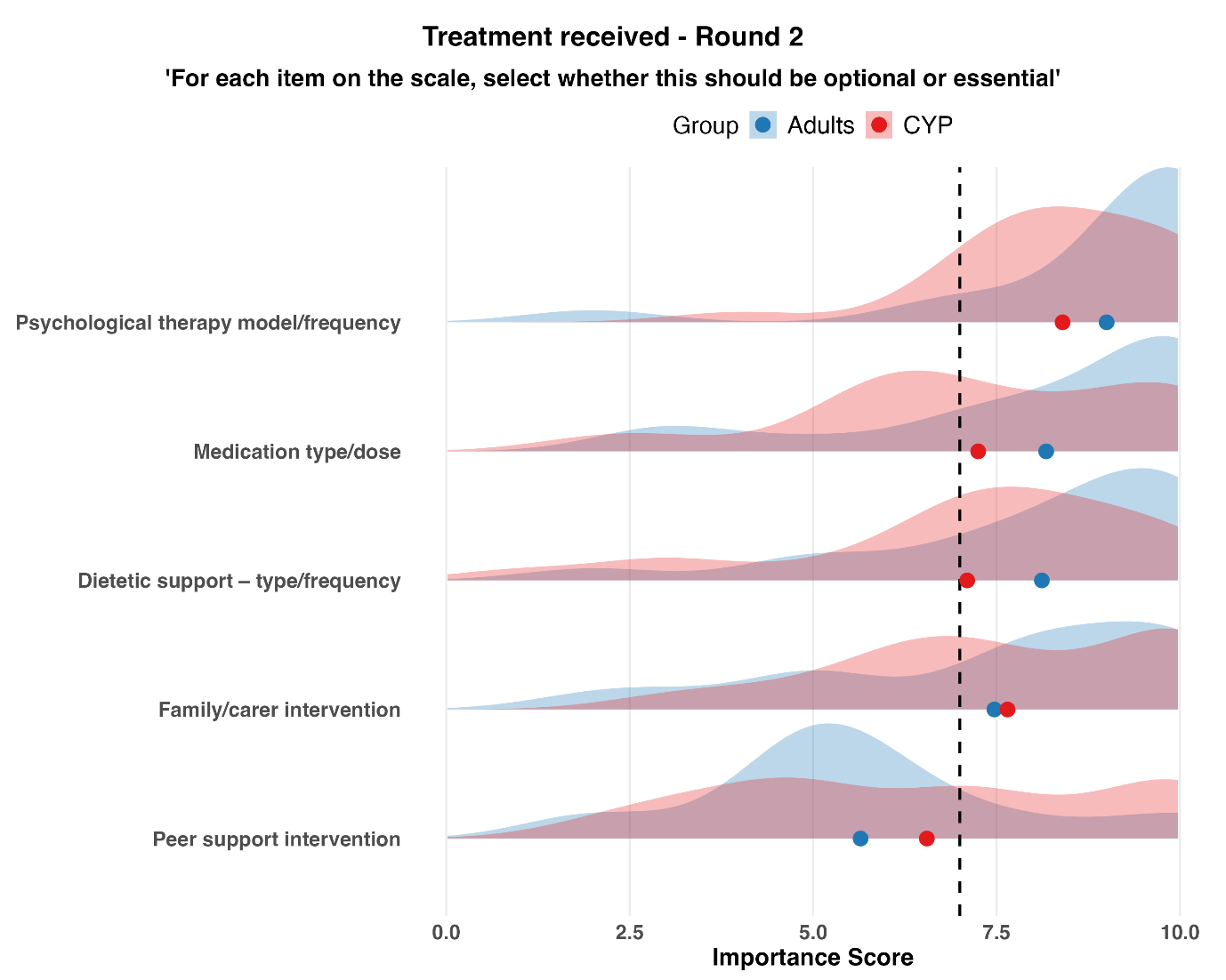


**Supplementary Figure 6: Voting on treatment received at Round 2 for Adult and Children/Young People workshops.**

Notes: CYP = Children and Young People.

**
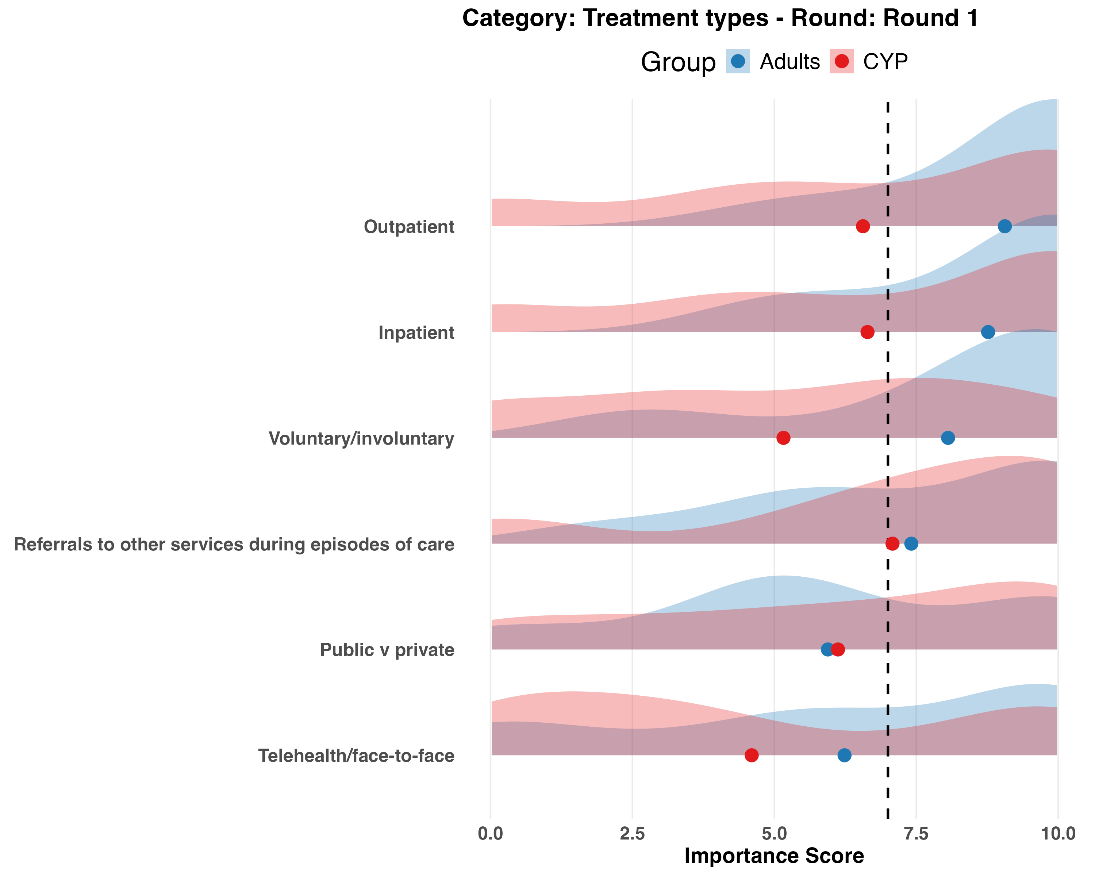
**

**Supplementary Figure 7: Voting on treatment type at Round 1 for Adult and Children/Young People workshops.**

Notes: CYP = Children and Young People.


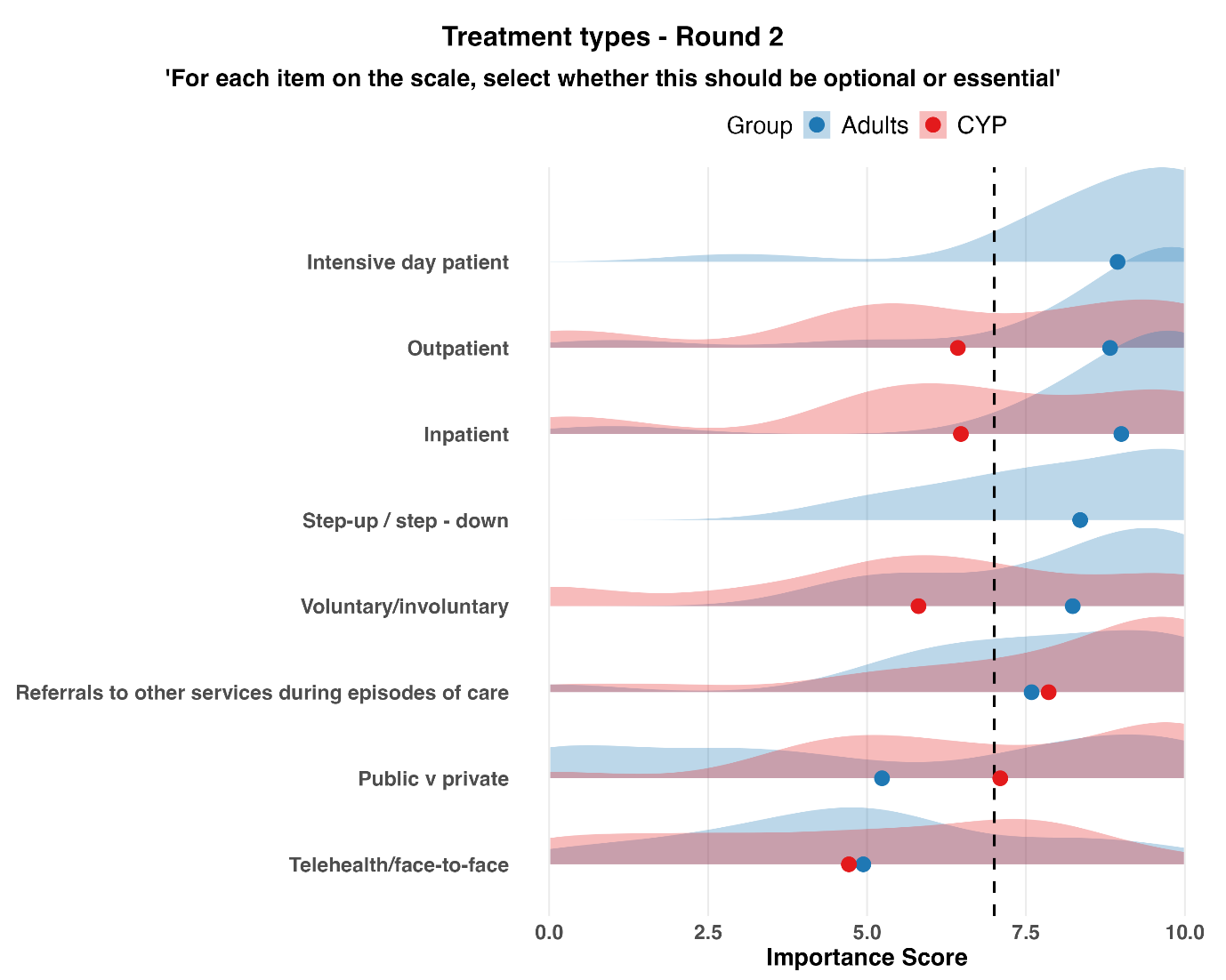


**Supplementary Figure 8: Voting on treatment type at Round 2 for Adult and Children/Young People workshops.**

Notes: CYP = Children and Young People.

**
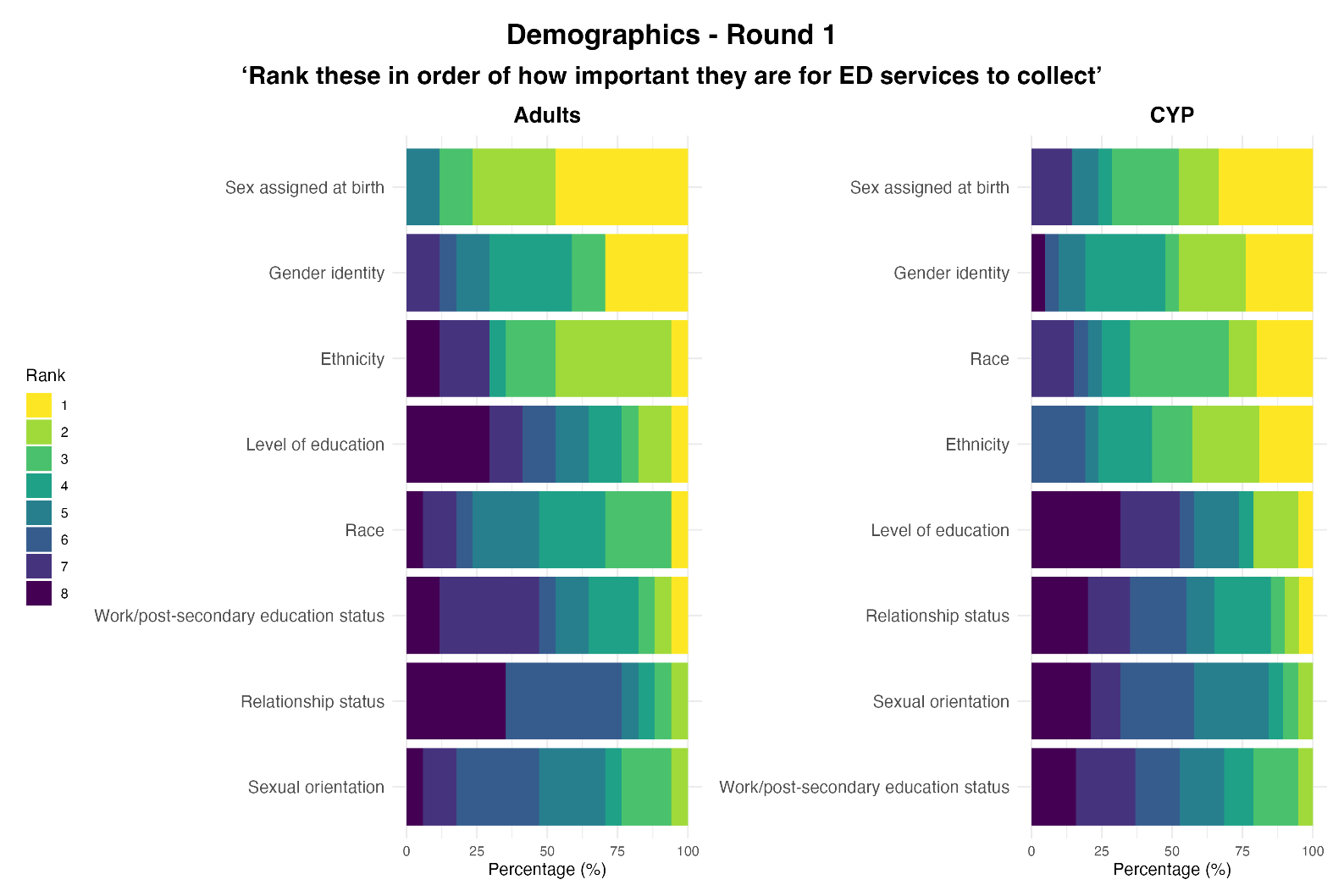
**

**Supplementary Figure 9: Ranking of demographic variables at Round 1 for Adult and Children/Young People workshops.**

Notes: Figure shows the variables in order of priority for the Adult and Children and Young People workshops, with highest priority displayed at the top. Within each bar, the coloured sections represent the percentage of participants who voted for that variable (e.g. the yellow section represents those voting for the variable as their first/highest priority). In the workshop voting, ‘sex assigned at birth’ was labelled as ‘gender’. CYP = Children and Young People.


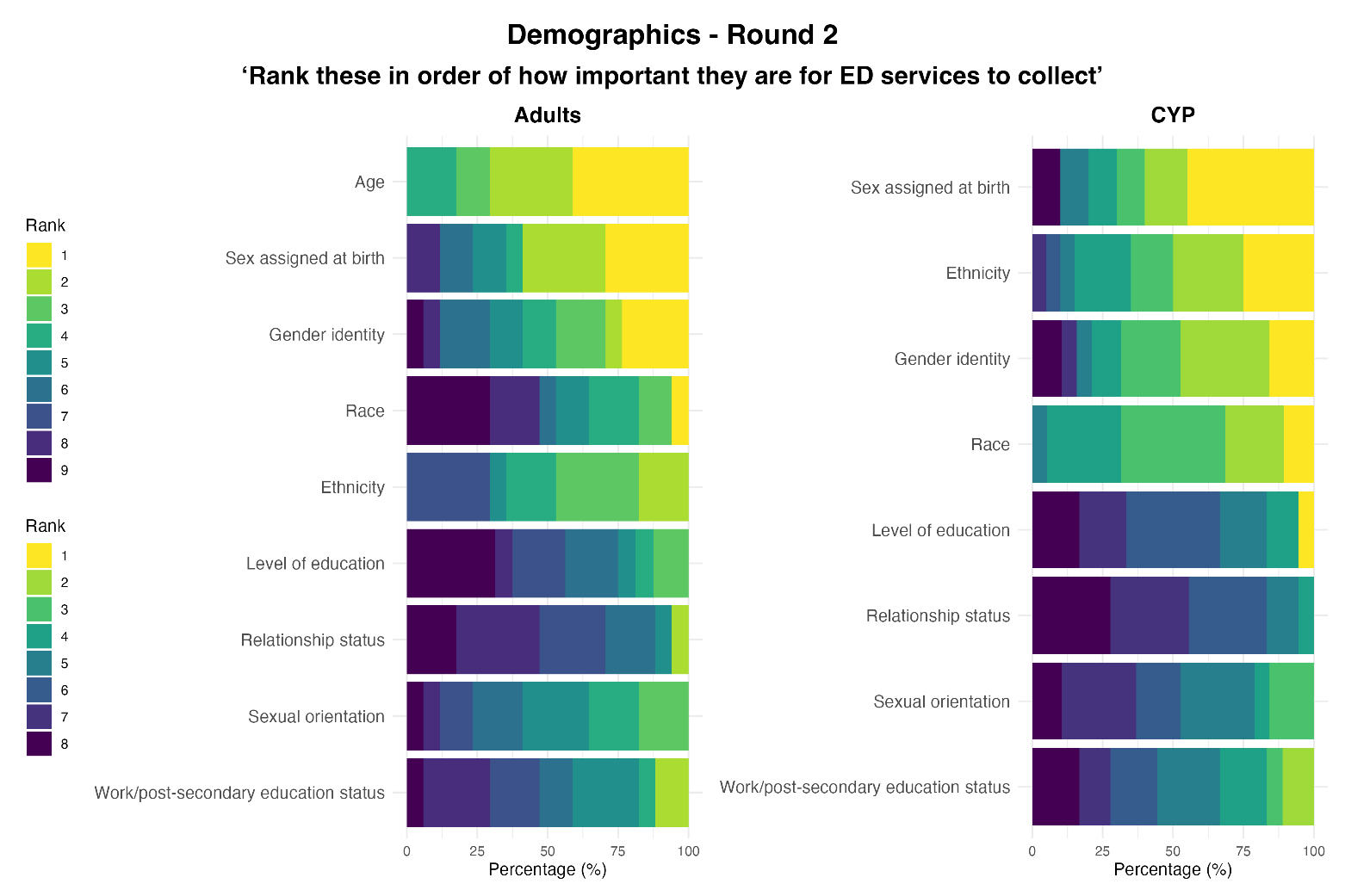


**Supplementary Figure 10: Ranking of demographic variables at Round 2 for Adult and Children/Young People workshops.**

Notes: Figure shows the variables in order of priority for the Adult and Children and Young People workshops, with highest priority displayed at the top. Within each bar, the coloured sections represent the percentage of participants who voted for that variable (e.g. the yellow section represents those voting for the variable as their first/highest priority). In the workshop voting, ‘sex assigned at birth’ was labelled as ‘gender’. CYP = Children and Young People.


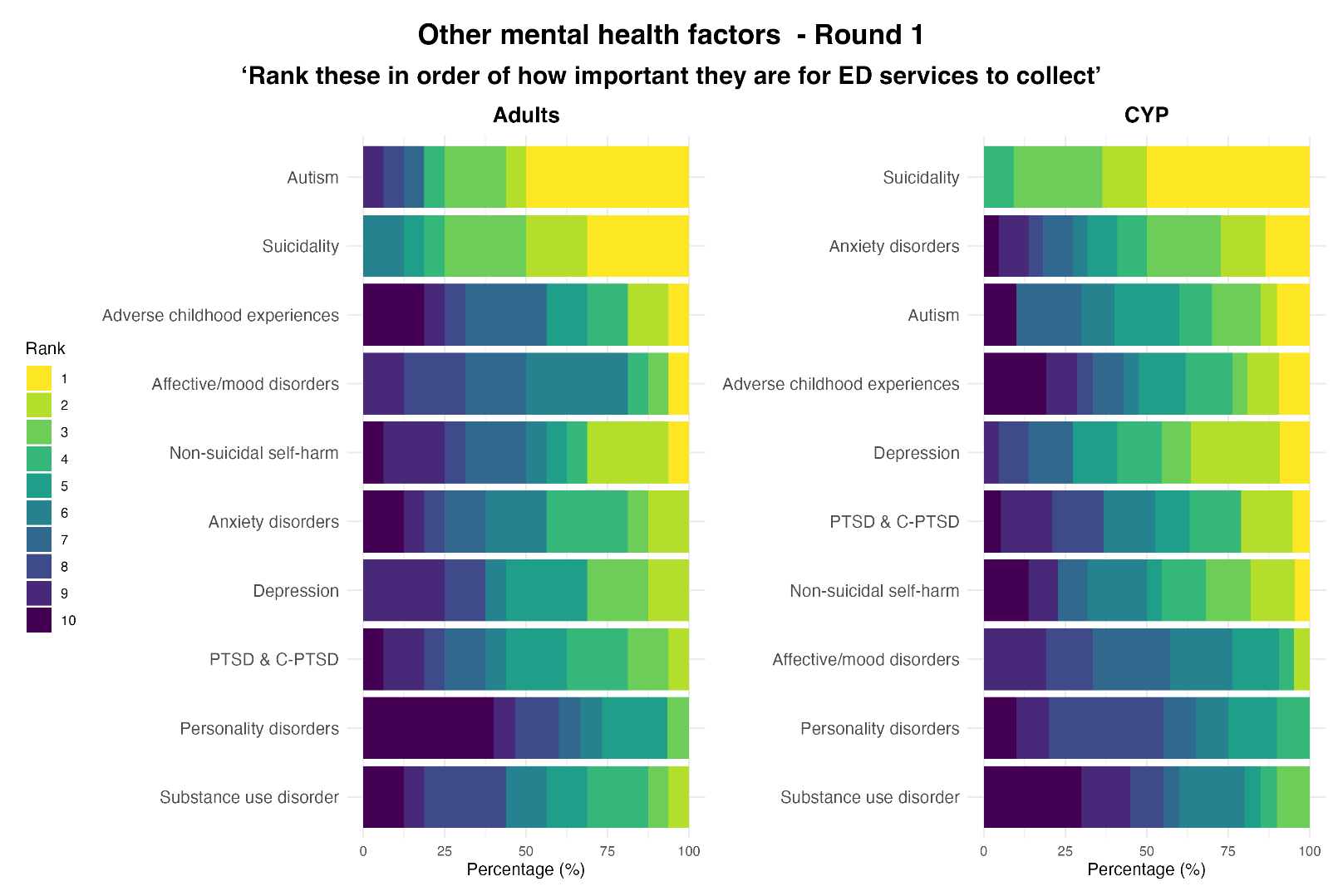


**Supplementary Figure 11: Ranking of other mental health and neurodevelopment factors at Round 1 for Adult and Children/Young People workshops.**

Notes: Figure shows the variables in order of priority for the Adult and Children and Young People workshops, with highest priority displayed at the top. Within each bar, the coloured sections represent the percentage of participants who voted for that variable (e.g. the yellow section represents those voting for the variable as their first/highest priority). CYP = Children and Young People.


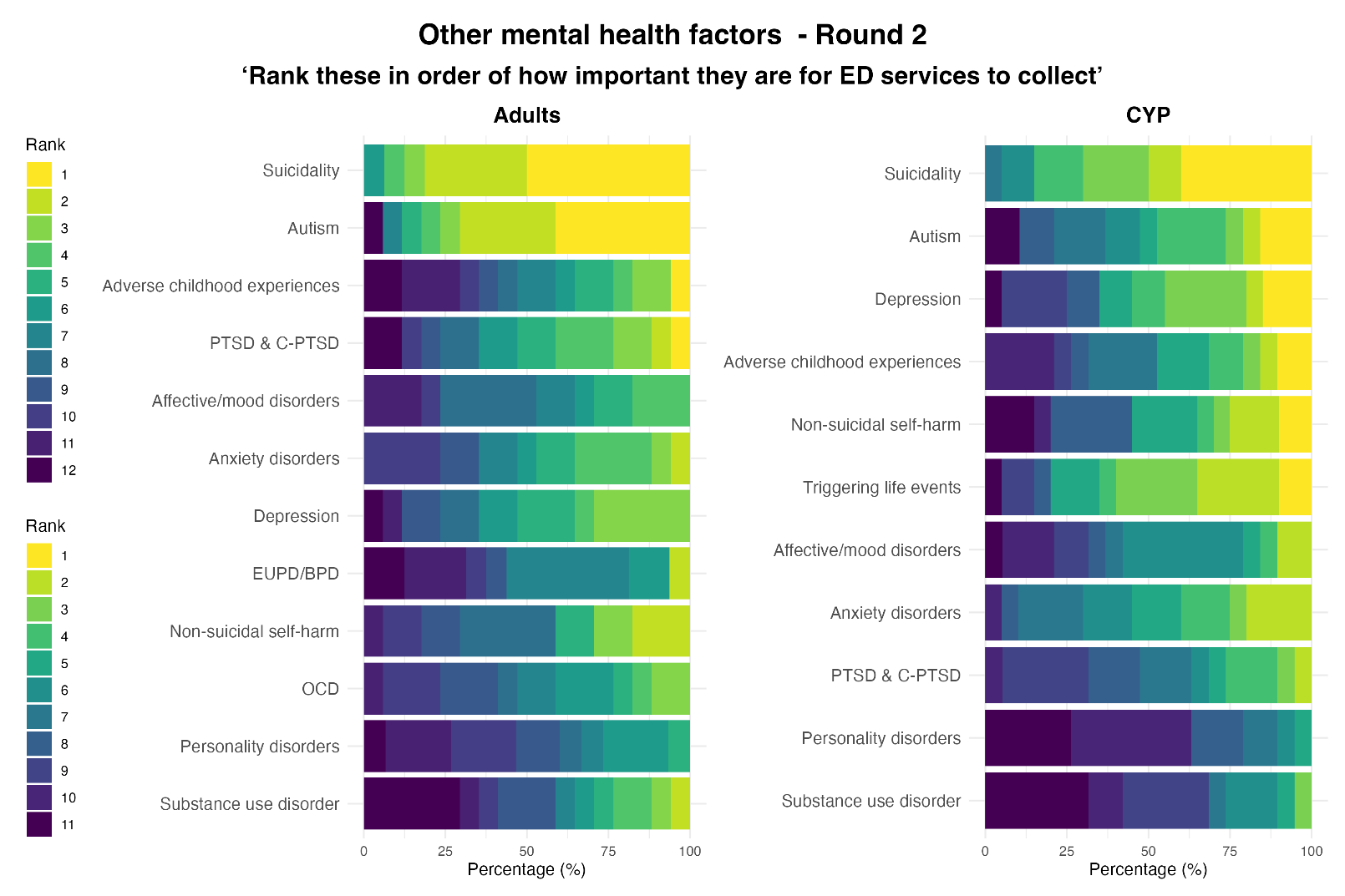


**Supplementary Figure 12: Ranking of other mental health and neurodevelopment factors at Round 2 for Adult and Children/Young People workshops.**

Notes: Figure shows the variables in order of priority for the Adult and Children and Young People workshops, with highest priority displayed at the top. Within each bar, the coloured sections represent the percentage of participants who voted for that variable (e.g. the yellow section represents those voting for the variable as their first/highest priority). CYP = Children and Young People.


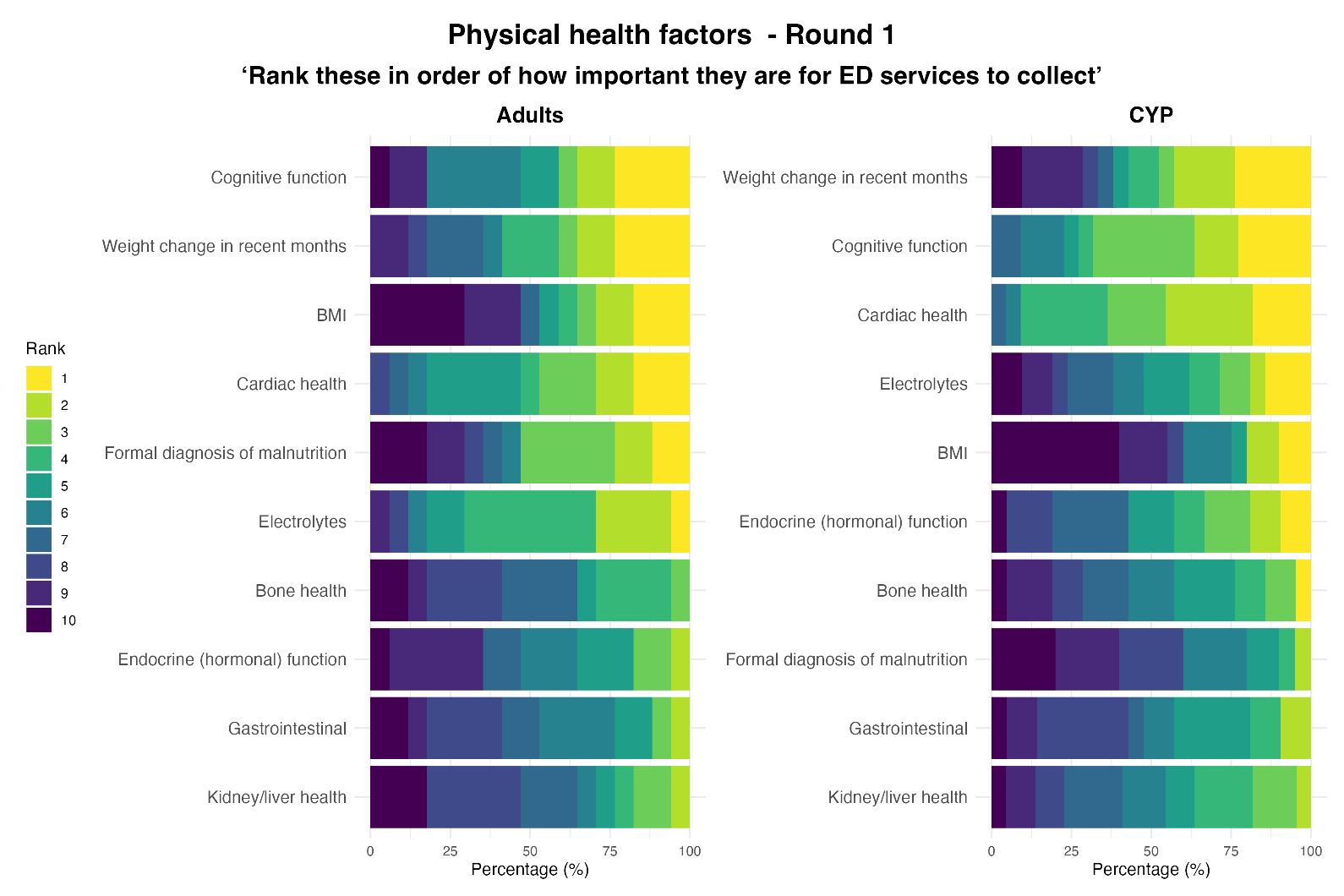


**Supplementary Figure 13: Ranking of physical health factors at Round 1 for Adult and Children/Young People workshops.**

Notes: Figure shows the variables in order of priority for the Adult and Children and Young People workshops, with highest priority displayed at the top. Within each bar, the coloured sections represent the percentage of participants who voted for that variable (e.g. the yellow section represents those voting for the variable as their first/highest priority). CYP = Children and Young People.


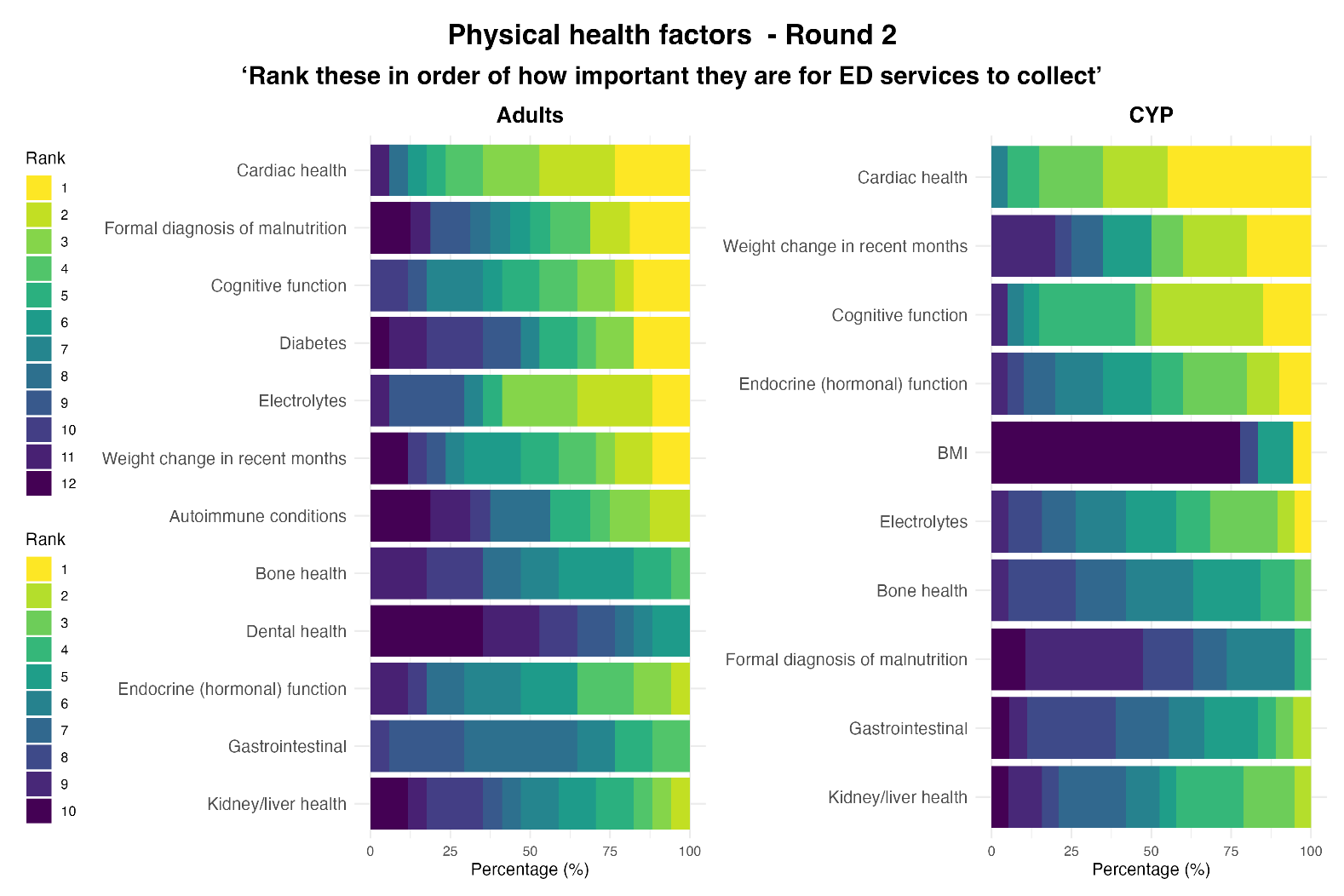


**Supplementary Figure 14: Ranking of physical health factors at Round 2 for Adult and Children/Young People workshops.**

Notes: Figure shows the variables in order of priority for the Adult and Children and Young People workshops, with highest priority displayed at the top. Within each bar, the coloured sections represent the percentage of participants who voted for that variable (e.g. the yellow section represents those voting for the variable as their first/highest priority). CYP = Children and Young People.


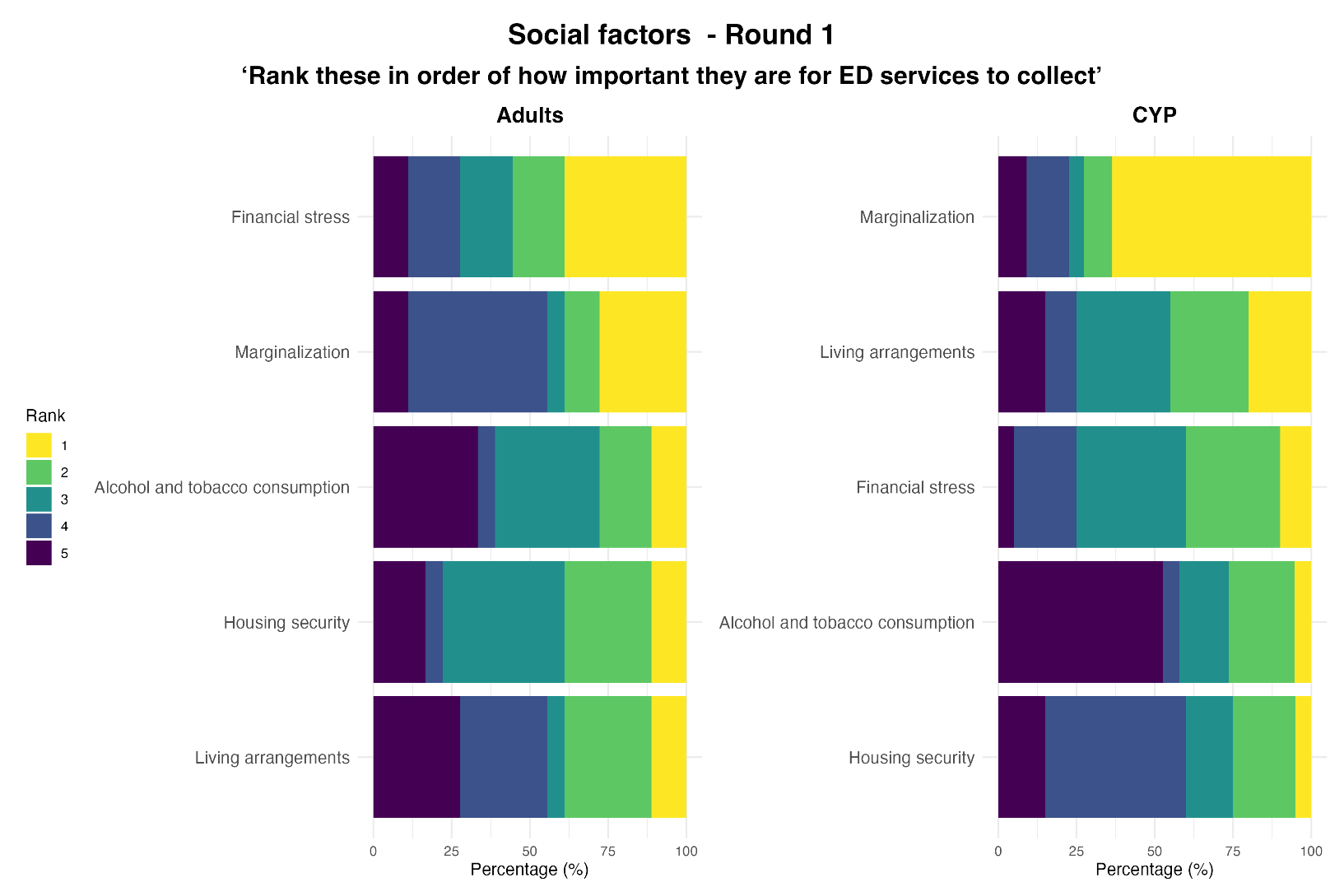


**Supplementary Figure 15: Ranking of social factors at Round 1 for Adult and Children/Young People workshops.**

Notes: Figure shows the variables in order of priority for the Adult and Children and Young People workshops, with highest priority displayed at the top. Within each bar, the coloured sections represent the percentage of participants who voted for that variable (e.g. the yellow section represents those voting for the variable as their first/highest priority). CYP = Children and Young People.


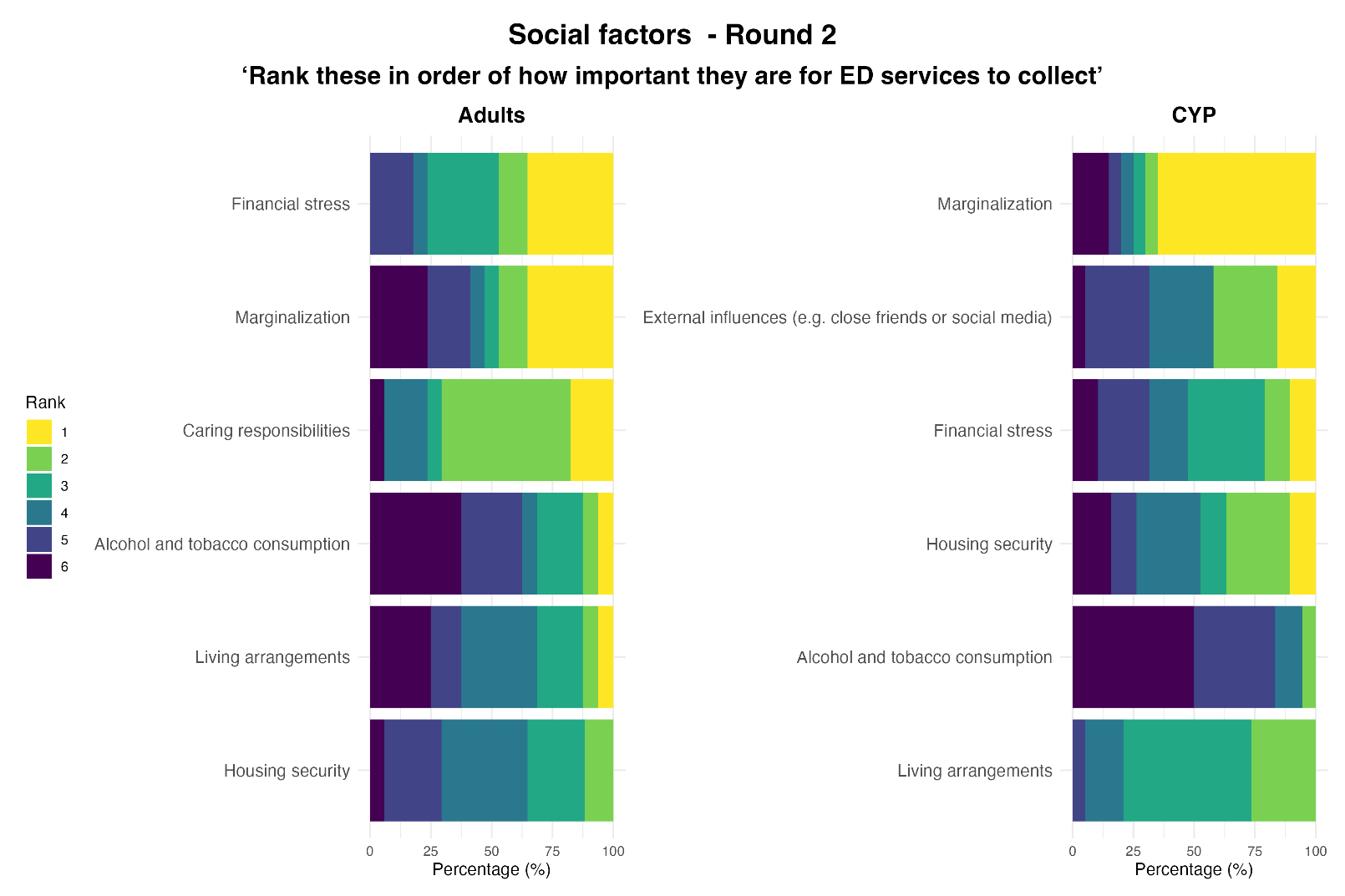


**Supplementary Figure 16: Ranking of social factors at Round 2 for Adult and Children/Young People workshops.**

Notes: Figure shows the variables in order of priority for the Adult and Children and Young People workshops, with highest priority displayed at the top. Within each bar, the coloured sections represent the percentage of participants who voted for that variable (e.g. the yellow section represents those voting for the variable as their first/highest priority). CYP = Children and Young People.

| **Supplementary 3: Thematic structure with additional illustrative quotes** | | |
| --- | --- | --- |
| **Themes** | **Sub-themes** | **Illustrative Quotes** |
| **A mutually valued and beneficial collaboration**  Participants emphasised that data collected by eating disorder services need to be both meaningful and of benefit to patients and other stakeholders. | **Addresses the challenges of treatment provision**  The need for data collected to speak to challenges in treatment provision, such as waiting times and treatment effectiveness. | *"The other thing I think’s quite important is the waiting time for treatment, because in my experience it seems to be the longer the wait is, the more severe symptoms tend to be by the time they get seen.” -* PWLE 7, Adult Workshop  *"In practice, person-centred care can be hard to implement due to systemic constraints and a lack of training for staff on how to genuinely engage in this approach."* - Carer 1, Adult Workshop  *"Patients should be given a voice in their treatment planning to ensure that their preferences and experiences are reflected in their care." -* PWLE 6, Adult Workshop  *“we did everything privately until the private section just unexpectedly said they cannot help anymore as this is too serious … They say that you have no other choice. So, I go to A&E … So, then we were on the bottom of everything again with regard to timing and knowledge. No one knew anything about us because we have been treated privately and I do not know if private and the NHS should work more together. I do not know if that could even be possible, but I am just trying to say that we were completely unknown. No one was interested in the notes from the private doctors and the private teams.” -* Carer 5, CYP Workshop  *“technically I am receiving treatment, but I am not fully receiving treatment … So, I have been with them for six months, so if we were looking at that as a start time for beginning treatment it is not really, they have just been touching base and linking in. So, there's questions as to when the actual start time is. Just because you may have accessed a service does not mean you have access to actual ongoing care for a variety of other reasons.” -* PWLE 2, CYP Workshop  *“we might have waited so long, and their conditions are getting worse and worse and worse because it is two different teams and they do not work together. It just does not make sense that we are talking about a human being who has all these aspects coexisting together. But as a treatment, they are two different teams.” -* Carer 5, CYP Workshop  *“People leave the treatment and you just don't know what happens to them. It's really hard to measure those outcomes … well, what happens if the people that actually leave the service actually end up recovering faster, is it because they're looking for other routes? … that's all lost in the data.” -* Carer 2, CYP Workshop  *"Because it seems like when you fill in these forms, it's just captured and it's inputted into that spreadsheet and it's all numbers and your data becomes a number. You're kind of ranked on your severity of your eating disorder, where there's no personal, “this is actually how severe it feels to me”. So clinically, it's not severe to you, but in your life it is severe because it has taken over every single day of your life and there needs to be some way of measuring that and putting in a personal perspective. So you don't just go “well, all these numbers mean this”” - PWLE 7, Adult Workshop*  *"We don’t have enough evidence really on what does and doesn’t work for different treatments based on other individual factors." -* PWLE 11, CYP Workshop  *“People leave the treatment and you just don't know what happens to them. It's really hard to measure those outcomes … well, what happens if the people that actually leave the service actually end up recovering faster, is it because they're looking for other routes? … that's all lost in the data.”* - Carer 2, CYP Workshop  *"Rural settings face significant resource constraints that impact the availability and quality of services compared to urban areas." -* Clinician 2, Adult Workshop  *“I think once, especially in CAMHS there's a lot of pigeonholing because it does make it easier to target treatment, but ultimately that doesn't really work for an eating disorders when eating disorders are as everyone's said, as fluid in their changing and yeah.” -* PWLE 5, CYP Workshop |
|  | **Viewed as worthwhile for everyone**  Data collected need to be perceived as meaningful and have the potential for positive impacts on patients, carers, providers, and the advancement of knowledge in the field. | *“Just recording our outcomes so that you can get paid by your commissioners is of value, but if it also helps you as a professional to risk profile the people in your care, it means that we can be so much more effective in the interventions we then take with individuals.” -* Clinician 3, CYP Workshop  *"The hardest thing about collecting data is getting your staff to understand what the value of the data is for. [...] It’s not just professionals that you have to convince to collect data; it’s also the service user because they are co-production partners with us in terms of collecting those data sets as well." - Clinician 3, CYP Workshop* *“Whereas if somebody was like, look, we keep this information because duh-duh-duh-duh-duh and kind of explained why it might be of benefit, A, to them and, B, to the ... outputs of the service in terms of research and bettering services and all the rest of it, then it seems that people are way more on board with filling out the paperwork and would maybe begrudge filling it in a lot less. And I think it's totally possible to give clients loads of paperwork to fill in if it's done in the right way.” -* Clinician 6, Adult Workshop  *“I suppose with my researcher hat on, the more data the better.” -* Clinician 6, Adult Workshop  *"[Clinician 3, CYP Workshop] raised the point about actually checking and challenging why we're collecting the data, what data we were collecting, and whether it is relevant, determining the frequency." -* Clinician 3, CYP Workshop  *"I wanted all the information, so it's very difficult for me to rate things lower. Cause I think all of it could be really important or interesting depending on the person." -* Clinician 5, Adult Workshop  *"People are generally happy to provide information, but it needs to be concise, and they need to know what’s being done with it. Regular updates or newsletters on how the data is used can keep participants engaged." -* Carer 2, CYP Workshop |
| **A holistic approach**  The importance of considering the whole person, including physical, psychological, and socio-cultural factors. | **Balances a range of measures**  The need to consider various factors and data points in treatment, recognising that everyone's journey and needs are unique. | *“I had other factors of trauma, physical disability, autism, and you know, if they weren't dealt with in the correct way, it made the motivation for change for eating disorder recovery much more difficult until they were addressed in a holistic way and bringing all those strands together and piecing them all up.” -* PWLE 5, Adult Workshop  *“I also think it’s important to include the broader picture, if there are other factors hindering an eating disorder diagnosis, then the holistic approach is key”* - PWLE 5, Adult Workshop  *“One of the things that we need to find out first of all is to collect all of this data and see where the relationships are and then do more with that data once we've got more of an idea where potential relationships between these different factors might exist.”* - Carer 4, CYP Workshop  *“DSM-5 does define the severity of restricting anorexia by BMI. So, you know, they have decided that BMI does have a place. MEED does use BMI as a measure of which traffic light you're on, so again, it has some place in restrictive anorexia. It doesn't have so much place in, for example, bulimia. You can have extreme bulimia and a normal weight, and extreme bulimia, as (participant name) pointed out, be very quickly fatal.”* - Carer 1, Adult Workshop  *"I felt that the questions were quite biased to restrictive eating disorders and that other eating disorders may not be screened very effectively through that questionnaire, which can be equally as serious. For example, very, very severe purging disorders can be, you know, fatal very quickly in certain circumstances when not picked up.”* - PWLE 6, Adult Workshop  *“When I was just reading those questions, they were made for anorexia … I have no idea what I would have really answered to those. So, I think that's something that it needs to be made maybe a bit more flexible”* - PWLE 9, Adult Workshop  *“I tend to disagree in motivation to change being something that’s important or high up on my factors and I think that’s because it can affect maybe how treatment is then perceived. I don’t think the motivation to change should necessarily affect the actual treatment that’s given … When I’ve been at a higher weight, I’ve had more motivation to change, not being at my worst.”* - PWLE 11, CYP Workshop  *“And I'm also a believer of positive factors. So, because we generally because we wanna do something about the risk factors and difficulties, et cetera, we sometimes over focus on difficult aspects. But I feel like it might be also important to try to get some information about the protective and supportive factors in people's life, because this is very valuable as well in terms of keeping them on track with the recovery or knowing that they will have some sort of support, ”* - Researcher 1, Adult Workshop |
|  | **Covers biopsychosocial domains & neurodivergence**  The importance of addressing biological, psychological, and social factors, as well as considering neurodivergence, in the treatment process. | *“Autism screening should be part of eating disorders and assessment. Personally, that's my feeling.”* - PWLE 3, Adult Workshop  *“It’s important to recognise how social factors contribute to eating disorders. For example, people who lack a support network are at higher risk of severe outcomes.”* - PWLE 8, Adult Workshop  *“I think the mental health comorbidities are massively important so are all health comorbidities. The treatment we have been through with our daughters, there's so much to address the eating disorder. But there's nothing to address any of the underlying stuff that must be dealt with completely separately, and yet they seem to go so hand in hand if you don't address”* - Carer 4, CYP Workshop  *"I think things like weight change in recent months is really useful."* - Carer 3, Adult Workshop  *"Social factors become really problematic in terms of, you know, if people are socially isolated, they might not have access to good support networks, which can contribute to the eating disorder. This creates a cycle of isolation that's very difficult to break."* - PWLE 8, Adult Workshop  *"I think for me it was really ‘What is their current experience of an eating disorder?’. So their behaviours, the thoughts, and the feelings that are going with it." -* PWLE 4, Adult Workshop  *"I was thinking of caring responsibilities or if there's any children in the home, maybe."* - Clinician 1, Adult Workshop  *"So I'm celiac, and I know there's a high link between celiacs and eating disorders and also diabetes. So they're all under autoimmune conditions and I think autoimmune conditions need to be screened alongside, with arthritis as well.”* - PWLE 7, Adult Workshop  *“Obviously cardiac function and electrolyte function just from a safeguarding perspective felt like the most important.”* - PWLE 4, Adult Workshop  *"I don't know this will come under bone health, but I do feel like eating disorders in all forms can really affect your teeth.” -* PWLE 4, Adult Workshop  *“we did talk about earlier about the family history, I find that one incredibly important and that should be there.”* - PWLE 9, Adult Workshop  *“Definitely anything like autism's very important and it's one of the things we're looking at in our team is how can we screen in eating disorder services.” -* PWLE 8, Adult Workshop  *“I was thinking you know on the list, so the social factors. I'm not sure whether this would come under living arrangements or maybe not, but I was thinking of caring responsibilities or if there's any children in the home, maybe.”* - Clinician 1, Adult Workshop  *"Possibly marginalisation is important to look at."* - Carer 3, Adult Workshop |
| **A balance between standardisation and individualisation**  The need to balance standardised data collection with the flexibility necessary to address individual differences in treatment. | **Creates standardised, comparable data**  The importance of standardised data collection to improve the quality of care and research as it promotes consistency and comparability across different settings. | *“When data collection isn't standardised, it not only affects the quality-of-care patients receive but also the quality of research that relies on that data. Without consistent data, it's hard to draw reliable conclusions or develop evidence-based practices that are truly applicable across different settings.” -* Clinician 1, Adult Workshop  *“I think the other thing is, and I know it's really difficult because we want to get consistent data for all of these different places, but one of the problems is that they all give you different information. I know that, for example, the meal plans that are given out at different places vary quite a lot. So, it's quite difficult to collect.”* - Carer 2, CYP Workshop  *"We can't just set a standard and forget about it. Data collection methods need to be continuously evaluated and updated to ensure they're still relevant, effective, and aligned with current best practices. This is an ongoing process, not a one-time fix."* - Carer 3, Adult Workshop  *"It's important to remember that standardisation doesn't mean one-size-fits-all. You have to adapt your data collection methods to fit the context, the culture, and the specific needs of the patient population you're working with. Otherwise, you risk collecting data that isn't relevant or accurate."* - PWLE 2, Adult Workshop  *“You have to adapt your data collection methods to fit the context, the culture, and the specific needs of the patient population you're working with. Otherwise, you risk collecting data that isn't relevant or accurate." -* PWLE 2, Adult Workshop |
|  | **Is flexible to include differences**  The need for flexibility in treatment to accommodate individual differences and unique experiences. | *"A broad open approach without trying to fit people into criteria [...] we’re all different. [...] There are probably some of us with big traumatic events. Other people may not have. [...] Even if it’s just a space on somebody's record like a notes box at the bottom." -* PWLE 10, CYP Workshop  *“Yeah, it absolutely feels like it can be completely fluid. So, although I was diagnosed with BED, I also did other different types. So, you know, we're in different patterns of eating at different times, one to negate the other, so to speak. So, you'd be jumping around the different ones.”* - PWLE 12, CYP Workshop  *“There’s so many routes to it. And what we were saying with the one size doesn’t fit all.” -* PWLE 5, CYP Workshop  *“I think everything else is really as important as each other, really. Yeah, bone health, cardiac. Again, it depends, doesn't it, how long the person has actually been entrenched in this illness; whether it's a first assessment for the first diagnosis and the first screening, or whether actually it's the 10th assessment and the 10th screening and however many years down the line.”* - Carer 3, Adult Workshop  *“I'd say that even though they are probably gonna end up with very similar diagnoses their journeys, their experiences, their motivators, they're all completely different and therefore my feeling was that we should collect all of these data because if everyone's journey is so individual, I don't think that we could collect something and not others. I don't necessarily think something is more important than another because it might be for that individual that one of these is just so crucial, and for somebody who's got the same diagnosis, something else is just so crucial.” -* Carer 4, CYP Workshop  *“with young adults with all various types of diabetes … there's type 1 disordered eating…so obviously with the what should be included were questions and assessments, it can be quite different with someone with a metabolic condition and therefore some of the questions and risk factors are a little bit different. And I just wondered if that's going to be considered and included with this.”* - Clinician 2, CYP Workshop  *"It's about, for me, people understanding the things that are most important to you that are impacting on your eating disorder and that's going to be different for everybody. So, it's really hard when you're ranking these things."* - PWLE 3, Adult Workshop  *“I think the questions must vary according to who you are and what you're going through at the time.”* - Carer 2, CYP Workshop |
| **Doing no harm**  The importance of ensuring that treatment processes do not cause harm by overburdening or stigmatising the patients. | **Is non-stigmatising and sensitive**  The need to build trust and reduce stigma by remaining sensitive and respectful when providing care. | *“Relationship building is key before jumping into questionnaires. It’s important to reduce shame and build trust, particularly for people who feel vulnerable.”* - PWLE 12, CYP Workshop  *“I've done something similar before, but it just doesn't always feel particularly personal. It feels very, again, number orientated, which I know is only a way clinically it can be taken down and analysed, but it doesn't feel particularly helpful.” -* PWLE 7, Adult Workshop  *“I think there's a danger in focusing too much on motivation to change. It can put a lot of pressure on the person going through it.”* - PWLE 10, CYP Workshop  *“where it said previous eating disorder and diagnoses and current eating diagnosis, I feel like the stress and importance placed on diagnosis can be more detrimental than helpful in targeting therapy.”* – PWLE 5, CYP Workshop  *"There's also an ethical dimension to data collection that we can't overlook. Patients have the right to know how their data is being used and to feel confident that it's being collected in a way that's respectful of their privacy and autonomy." -* Carer 3, Adult Workshop  *“Well, in terms of being an adult, that question is incredibly judgment loaded. And if you have something like BED, you already judge yourself on a very regular basis. So, your motivation is always there but you just can’t do anything about it, you feel powerless. And so actually that question is a loaded, powerful question, but it’s power over the patient. And I have a big reaction to that.”* - PWLE 12, CYP Workshop  *“When people have a longer duration of illness that bad experiences with treatment, not only hamper your motivation for engaging in further treatment, but they can be quite traumatic, so then you've got these additional barriers when you then seek further treatment, because not only do you have all the difficulties you've first presented to treatment with, but you've got the difficulty of needing to rebuild trust again in services.”* - PWLE 8, Adult Workshop  *"It’s important to know what the information is being used for and ensure feedback is given. Without understanding the purpose, people may feel overwhelmed and disengaged."* - PWLE 11, CYP Workshop  *"If you can build a relationship where the patient feels understood and not judged, it can make a huge difference in their motivation to engage in treatment. That trust can be the foundation for change, even in patients who have been labelled as 'difficult' or 'resistant' in the past." -* Clinician 1, Adult Workshop  *"Creating an environment where patients feel safe and understood is fundamental." -* PWLE 6, Adult Workshop |
|  | **Does not overburden**  The importance of avoiding high levels of participant burden. | *“How do we assess what’s most relevant without overburdening people with too many measures? We need a balance between gathering essential data and not overwhelming the patient.”* - Clinician 3, CYP Workshop  *“it seems to me that for the most part, cause of the time constraints in statutory settings versus the luxuries that we have in private practice, the clients often have no comprehension, because it wasn't explained to them”* - Clinician 6, Adult Workshop  *“the clients often have no comprehension, because it wasn't explained to them, why they're filling in forms and it just feels often to them that they’re given a form to fill in rather than somebody caring enough to ask the questions.”* - Clinician 6, Adult Workshop  *“... one of my worries is that this would be doubling up. So, you know, if people are being asked to answer all of these things as questionnaire measures, you know, in electronic ways that is available to everyone, which is absolutely fantastic and definitely what we need. But then the clinicians might need to ask it all again to get more detail around it or more nuance and the context and things. And that's gonna be really frustrating possibly for people and upsetting to have to go through it all again. And I guess the other thing was I wanted all the information, so it's very difficult for me to rate things lower.”* - Clinician 5, Adult Workshop  *“when we ask people to fill out so many questionnaires, in research and in clinical setting, it's too much. Of course, we wanna have objective measures and when we give the rationale quite nicely, people wanna do, but at the same time it's really taking so much time and they may not have energy to do this or time, etcetera.”* - Researcher 1, Adult Workshop |

| **Supplementary 4: Optional self-report measures** | | | |
| --- | --- | --- | --- |
| **Variable** | **Timepoint(s)** | **Reporter** | **Tool** |
| ***Optional:*** Physical/somatic symptoms | Assessment, Start of regular treatment (if >1 month later than assessment), 3 monthly while in treatment, Transition, if applicable (from IP to DP etc.), Discharge, ***Optional follow-up, if patient consents to being contacted after discharge*** | Patient | [PHQ-15](https://emckclac.sharepoint.com/:b:/r/sites/MT-EDCRN/Shared%20Documents/General/Minimum%20dataset/Questionnaires/PHQ-15.pdf?csf=1&web=1&e=qBFaCw) (Adult 18+) |
| ***Optional:*** ADHD | Assessment, Discharge | Patient | [ASRS](https://emckclac.sharepoint.com/:b:/r/sites/MT-EDCRN/Shared%20Documents/General/Minimum%20dataset/Questionnaires/Adult%20ADHD%20Self-Report%20Scale%20(ASRS).pdf?csf=1&web=1&e=565Nrm) (Adult/child aged 13+) |
| ***Optional:*** Psychosocial impairment | ***Optional follow-up, if patient consents to being contacted after discharge*** | Patient | [EQ-5D-5L](https://emckclac.sharepoint.com/:b:/r/sites/MT-EDCRN/Shared%20Documents/General/Minimum%20dataset/Questionnaires/EQ-5D-5L.pdf?csf=1&web=1&e=i6FY6C) (adult 18+)  [KIDSCREEN-10](https://emckclac.sharepoint.com/:b:/r/sites/MT-EDCRN/Shared%20Documents/General/Minimum%20dataset/Questionnaires/KIDSCREEN-10%20index_ChildrenAdolescents_UK.pdf?csf=1&web=1&e=hEevSC) (child – 8-17yrs) |
| ***Optional:*** Alcohol use | Assessment | Patient | [AUDIT-C](https://emckclac.sharepoint.com/:b:/r/sites/MT-EDCRN/Shared%20Documents/General/Minimum%20dataset/Questionnaires/Alcohol_use_disorders_identification_test__AUDIT_.pdf?csf=1&web=1&e=usw82E) (Adult 18+) and [CAGE](https://emckclac.sharepoint.com/:b:/r/sites/MT-EDCRN/Shared%20Documents/General/Minimum%20dataset/Questionnaires/CAGE_questionaire.pdf?csf=1&web=1&e=B985at) (Adult 18+) |
| ***Optional:*** PTSD symptoms (inc. dissociation) | Assessment | Patient | [PTSD Checklist-6](https://emckclac.sharepoint.com/:b:/r/sites/MT-EDCRN/Shared%20Documents/General/Minimum%20dataset/Questionnaires/PTSD%20Checklist%206.pdf?csf=1&web=1&e=a70tkB) (Adult 18+)  [CTS-5](https://emckclac.sharepoint.com/:b:/r/sites/MT-EDCRN/Shared%20Documents/General/Minimum%20dataset/Questionnaires/CTS.pdf?csf=1&web=1&e=Deve8S) (Child 8-17yrs)  and/or  [CPSS-5](https://emckclac.sharepoint.com/:b:/r/sites/MT-EDCRN/Shared%20Documents/General/Minimum%20dataset/Questionnaires/cpss-5.pdf?csf=1&web=1&e=ZS4hxa) (Child 8-17yrs) |
| ***Optional:*** Self-administered comorbidity | Assessment, Admission, Transition (from IP to DP etc.), Discharge, ***Optional follow-up, if patient consents to being contacted after discharge*** | Patient | [SCQ](https://emckclac.sharepoint.com/:i:/r/sites/MT-EDCRN/Shared%20Documents/General/Minimum%20dataset/Questionnaires/SCQ.jpg?csf=1&web=1&e=btZvHh) (Adult 18+) – separate MH, ED & Physical |
| ***Optional:*** Weight stigma | Assessment, Admission, Transition (from IP to DP etc.), Discharge, ***Optional follow-up, if patient consents to being contacted after discharge*** | Patient | Items 3,5,6 of the [WBIS-M](https://emckclac.sharepoint.com/:b:/r/sites/MT-EDCRN/Shared%20Documents/General/Minimum%20dataset/Questionnaires/WBIS-M.pdf?csf=1&web=1&e=4PKk3y) (18+)  ***Optional [for people in larger bodies]:*** [WBIS](https://emckclac.sharepoint.com/:w:/r/sites/MT-EDCRN/Shared%20Documents/General/Minimum%20dataset/Questionnaires/WBIS-11-item.doc?d=w73971b4225134677b213014fde1d7c57&csf=1&web=1&e=oOlej2) (18+)  And:  ***Optional [for people in larger bodies]:*** [STEWS](https://emckclac.sharepoint.com/:w:/r/sites/MT-EDCRN/Shared%20Documents/General/Minimum%20dataset/Questionnaires/STEWS.docx?d=w219c7fb817ec4b868393536eefc9bebb&csf=1&web=1&e=g4hGYu) (18+) |
| ***Optional:*** Treatment experience | ***Optional at discharge*** | Patient | [Adult/child](https://emckclac.sharepoint.com/:w:/r/sites/MT-EDCRN/Shared%20Documents/General/Minimum%20dataset/Questionnaires/Treatment%20experience.docx?d=wc7f1a0954b4c4de2bbbeaf8b8295a6a3&csf=1&web=1&e=zl5si4) (16-+) |
| ***Optional:*** Mental health comorbidities | Assessment | Clinician | Via the provisional problems list of the [Current View Tool (29)](https://emckclac.sharepoint.com/:b:/r/sites/MT-EDCRN/Shared%20Documents/General/Minimum%20dataset/Questionnaires/Current%20view%20tool.pdf?csf=1&web=1&e=joHXZC) |
| ***Optional:*** Externalised weight stigma | Assessment | Clinician | [F-Scale short-form](https://emckclac.sharepoint.com/:i:/r/sites/MT-EDCRN/Shared%20Documents/General/Minimum%20dataset/Questionnaires/F-Scale%20short%20form.png?csf=1&web=1&e=pcv3cU) |
| ***Optional:*** Caregiver-reported measures - information | Admission | Parent/guardian | [CaNAM](https://emckclac.sharepoint.com/:b:/r/sites/MT-EDCRN/Shared%20Documents/General/Minimum%20dataset/Questionnaires/CaNAM.pdf?csf=1&web=1&e=0vjTlH) |
| ***Optional:*** Caregiver-reported measures – Confidence and impact | 3 months into treatment, Discharge | Parent/Guardian | [EDSIS](https://emckclac.sharepoint.com/:b:/r/sites/MT-EDCRN/Shared%20Documents/General/Minimum%20dataset/Questionnaires/EDSIS.pdf?csf=1&web=1&e=RdM7vK) and [PACCS](https://emckclac.sharepoint.com/:b:/r/sites/MT-EDCRN/Shared%20Documents/General/Minimum%20dataset/Questionnaires/PACCS.pdf?csf=1&web=1&e=CojPSl) |
| Note. ASRS = Adult ADHD Self-Report Scale; EQ-5D-5L = Health Related Quality of Life measure; AUDIT-C = Alcohol Use Disorders Identification Test - Consumption; CAGE = 4-item questionnaire to screen for alcohol misuse; CTS-5 = Childhood Trauma Screener - 5 item; CPSS-5 = Child PTSD Symptom Scale for DSM-5; SCQ = Self-Administered Co-morbidities questionnaire; MH = mental health; WBIS-M = Weight Bias Internalization Scale - Modified; WBIS = Weight Bias Internalization Scale; STEWS = Scale for Treatment-based Experiences of Weight Stigma; F-Scale = Fat Phobia Scale; CaNAM = Carers Needs Assessment Measure; EDSIS = Eating Disorders Symptom Impact Scale; PACCS = Patient and Carer Collaboration Scale | | | |

# **Supplementary 5: Demographics and ED history Questionnaire – patients**

What is your gender identity?

- Male
- Female
- Non-binary
- Trans woman
- Trans man
- Other gender identity (please write)
- Prefer not to answer

Is this the same as your sex assigned at birth?

- Yes
- No
- Prefer not to answer

What is your ethnicity?

- Not known
- White – British
- White – Irish
- White – any other White background
- Mixed – White and Black Carribbean
- Mixed – White and Black African
- Mixed – White and Asian
- Mixed – any other Mixed background
- Asian or Asian British – Indian
- Asian or Asian British – Pakistani
- Asian or Asian British – Bangladeshi
- Asian or Asian British – Any other Asian background
- Black or Black British – Caribbean
- Black or Black British – African
- Black or Black British – Any other Black background
- Other Ethnic Groups – Chinese
- Other Ethnic Groups – Any other ethnic group
- Prefer not to answer

What is the highest level of education you have completed?

- Some primary
- Completed primary
- Some secondary
- Completed secondary
- Vocational or similar
- Some university but no degree
- University Bachelors Degree
- Graduate or professional degree (e.g. MA, MS, MBA, PhD, JD, MD, DDS)
- Other
- Prefer not to say

What is your current employment status?

- Fulltime employment
- Part-time employment
- Self-employed
- Unemployed
- Retired
- Looking after home/family
- Unable to work due to sickness or disability
- Doing unpaid or voluntary work
- Full-time or part-time student
- Other
- Prefer not to say

What are your living arrangements?

- Lives in privately owned property
- Living in privately rented accommodation
- Living in social rented accommodation
- Living in student accommodation
- Other
- Prefer not to say

What country do you live in?

- England
- Scotland
- Wales
- Nothern Ireland
- Other (please specify)

What is your postcode?

Do you look after or give any help or support to, anyone because they have long-term physical or mental health conditions or illnesses, or problems related to old age?

- No
- Yes, 9 hours a week or less
- Yes, 10-19 hours a week
- Yes, 20-34 hours a week
- Yes, 35-49 hours a week
- Yes, 50 or more hours a week

What is your current relationship status?

- Single
- Relationship (not living together)
- Relationship (living together)
- Married or civil partnership
- Separated
- Divorced
- Widowed
- Prefer not to answer

Which of the following best describes your sexual orientation?

- Straight or Heterosexual
- Gay or Lesbian
- Bisexual
- Pansexual
- Asexual
- Queer
- Other
- Prefer not to say

What age did your eating disorder develop?

What previous treatment have you received for your eating disorder? *Please select all that apply.*

- None
- Outpatient
- Day-patient
- Inpatient
- Non-specialist eating disorder support
- Other

Which of the following eating disorders do you have? *Please select all that apply.*

- Anorexia Nervosa
- Bulimia Nervosa
- Binge Eating Disorder
- Avoidant Restrictive Food Intake Disorder (ARFID)
- Atypical Anorexia Nervosa
- Purging Disorder
- Night eating syndrome
- Other eating disorder
- Don’t know
- Prefer not to answer

Is there a history of eating disorders in your immediate family?

- Yes
- No
- Don’t know
- Prefer not to answer

Is there a history of other mental health problems in your immediate family?

- Yes
- No
- Don’t know
- Prefer not to answer

# **Supplementary 6: Demographics and ED history Questionnaire – caregivers**

What is your gender identity?

- Male
- Female
- Non-binary
- Trans woman
- Trans man
- Other gender identity (please write)
- Prefer not to answer

Is this the same as your sex assigned at birth?

- Yes
- No
- Prefer not to answer

What is your ethnicity?

- Not known
- White – British
- White – Irish
- White – any other White background
- Mixed – White and Black Carribbean
- Mixed – White and Black African
- Mixed – White and Asian
- Mixed – any other Mixed background
- Asian or Asian British – Indian
- Asian or Asian British – Pakistani
- Asian or Asian British – Bangladeshi
- Asian or Asian British – Any other Asian background
- Black or Black British – Caribbean
- Black or Black British – African
- Black or Black British – Any other Black background
- Other Ethnic Groups – Chinese
- Other Ethnic Groups – Any other ethnic group
- Prefer not to answer

What is the highest level of education you have completed?

- Some primary
- Completed primary
- Some secondary
- Completed secondary
- Vocational or similar
- Some university but no degree
- University Bachelors Degree
- Graduate or professional degree (e.g. MA, MS, MBA, PhD, JD, MD, DDS)
- Other
- Prefer not to say

What is your current employment status?

- Fulltime employment
- Part-time employment
- Self-employed
- Unemployed
- Retired
- Looking after home/family
- Unable to work due to sickness or disability
- Doing unpaid or voluntary work
- Full-time or part-time student
- Other
- Prefer not to say

What are your living arrangements?

- Lives in privately owned property
- Living in privately rented accommodation
- Living in social rented accommodation
- Living in student accommodation
- Other
- Prefer not to say

What country do you live in?

- England
- Scotland
- Wales
- Northern Ireland
- Other (please specify)

What is your postcode?

Do you look after or give any help or support to, anyone because they have long-term physical or mental health conditions or illnesses, or problems related to old age?

- No
- Yes, 9 hours a week or less
- Yes, 10-19 hours a week
- Yes, 20-34 hours a week
- Yes, 35-49 hours a week
- Yes, 50 or more hours a week

What age did your loved one’s eating disorder develop?

What previous treatment has your loved one received for their eating disorder? *Please select all that apply.*

- None
- Outpatient
- Day-patient
- Inpatient
- Non-specialist eating disorder support
- Other

Which of the following eating disorders does your loved one have? *Please select all that apply.*

- Anorexia Nervosa
- Bulimia Nervosa
- Binge Eating Disorder
- Avoidant Restrictive Food Intake Disorder (ARFID)
- Atypical Anorexia Nervosa
- Purging Disorder
- Night eating syndrome
- Other eating disorder
- Don’t know
- Prefer not to answer

Do you, or have you ever had an eating disorder (whether or not you received a diagnosis)?

- Yes – currently
  - Please specify which Eating Disorder(s)
- Yes – in the past
  - Please specify which Eating Disorder(s)
- No
- Prefer not to answer

[IF YES] Has this condition developed, worsened or returned while caring for your loved one with an eating disorder?

- Yes – developed
- Yes – worsened
- Yes – returned
- No
- Prefer not to say

Do you, or have you ever had any other mental health condition (whether or not you received a diagnosis)?

- Yes – currently
  - Please specify which mental health condition(s)
- Yes – in the past
  - Please specify which mental health condition(s)
- No
- Prefer not to answer

[IF YES] Has this condition developed, worsened or returned while caring for your loved one with an eating disorder?

- Yes – developed
- Yes – worsened
- Yes – returned
- No
- Prefer not to say

Is there a history of eating disorders in your immediate family?

- Yes
- No
- Don’t know
- Prefer not to answer

Is there a history of other mental health problems in your immediate family?

- Yes
- No
- Don’t know
- Prefer not to answer

# **Supplementary 7: Clinician and service reported variables**

**Clinician/Service reported information at assessment:**

- Eating disorder diagnosis Date of referral
- Date of assessment
- Estimated month and year of first meeting criteria for a *diagnosable* eating disorder
- Estimated month and year of first experiencing any eating disorder symptoms
- Is the patient being seen through FREED (First Episode Rapid Early Intervention for Eating Disorders)?
  - No
  - Yes, assessed through FREED but not FREED eligible
  - Yes, assessed through FREED and is FREED eligible
    - Please enter Duration of Untreated Eating Disorder (DUED; time in months between onset of a diagnosable eating disorder and date of assessment)
- Physical health information – summarised elsewhere (height/weight/BMI/blood tests):
  - Weight history
    - Highest weight as an adult
    - Lowest weight as an adult
  - BMI
    - Weight – KG OR St and Lbs
    - Height – CM OR Ft and In.
  - Muscle power
    - SUSS (Sit Up Squat Stand test) score
    - HGS (Hand grip strength) score
  - Blood test results
    - See list 1-7 in EDCRN dataset
- Treatment plan using drop-down options, with multiple options able to be selected:
  - Inpatient
    - Specialist EDU inpatient treatment
    - General medical ward inpatient treatment
    - General psychiatric ward inpatient treatment
  - Day-services
    - Full-time EDU day-services programme (9-5 Mon-Fri)
    - Part-time EDU day-services programme (less than 9-5 Mon-Fri):
      - *[open text] Add more detail if desired (e.g. number of days):*
    - Non-eating disorder focused day-services care
  - Intensive outreach, enhanced treatment team or home treatment team treatment
    - Eating disorder focused intensive outreach treatment
    - Non-eating disorder focused intensive outreach treatment
  - Outpatient psychological therapy – select from
    - Guided self-help based on CBT
    - CBT-T (10-session CBT for eating disorders)
    - CBT-ED/CBT-E (CBT for eating disorders / Enhanced CBT for eating disorders
    - CBT for ARFID
    - MANTRA (Maudsley model Anorexia Nervosa Treatment for Adults)
    - SSCM (Specialist Supportive Clinical Management)
    - FT-AN / FT-BN / FBT (Family based treatment for eating disorders)
    - Other
      - Focal psychodynamic psychotherapy
      - Cognitive analytical therapy
      - Schema therapy
      - Dialectical behaviour therapy
      - Radically open dialectical behaviour therapy
      - Other, please specify
  - After selecting outpatient therapy, to select if individual or group or a mix of individual + group
  - Other outpatient support
    - Dietetics
    - Occupational therapy
    - Family therapy (not specifically focused on the eating disorder)
    - Carer support sessions or carer skills workshops
    - Peer support
    - Psychiatry or medical reviews
    - Nursing or physical health monitoring reviews
    - Nutritional supplements or prescriptions (please only select medications that were recommended or prescribed as part of eating disorder team input)
      - Multivitamin
      - Potassium supplements (e.g., Sando-K)
      - Magnesium supplements
      - Phosphate supplements
      - Calcium supplements
      - Iron/ferritin supplements
      - Vitamin D supplements
      - Other (please specify)
    - Psychotropic medication (please only select medications that were recommended or prescribed as part of eating disorder team input)
      - Antidepressants
        - SSRI antidepressants (e.g., citalopram, escitalopram, fluoxetine, paroxetine, sertraline)

Please provide type and dose if known

- - - - - SNRI antidepressants (e.g., atomoxetine, duloxetine, venlafaxine)

Please provide type and dose if known

- - - - - NASSA antidepressants (e.g., mirtazapine)

Please provide type and dose if known

- - - - - Tricyclic antidepressants (e.g., amitriptyline)

Please provide type and dose if known

- - - - - Other antidepressant

Please provide type and dose if known

- - - - Antipsychotic / atypical antipsychotic (e.g., olanzapine)
        - Please provide type and dose if known
      - Anxiolytics / anti-anxiety / insomnia / sedative medications
        - Anaesthetics (e.g., ketamine, propofol)

Please provide type and dose if known

- - - - - Antihistamines (e.g., diphenhydramine, promethazine)

Please provide type and dose if known

- - - - - Barbiturates (e.g., amobarbital, butalbital)

Please provide type and dose if known

- - - - - Benzodiazepines (e.g., diazepam, lorazepam, temazepam)

Please provide type and dose if known

- - - - - Buspirone

Please provide type and dose if known

- - - - - Melatonin

Please provide type and dose if known

- - - - - Muscle relaxants (e.g., clonidine, pregabalin)

Please provide type and dose if known

- - - - - Non-benzodiazepine hypnotics (e.g., zolpidem, zopiclone)

Please provide type and dose if known

- - - - - Other anti-anxiety or sedative medications

Please provide type and dose if known

- - - - Mood stabilisers (non antipsychotic)
        - Anticonvulsants (e.g. carbamazepine, lamotrigine)

Please provide type and dose if known

- - - - - Lithium

Please provide type and dose if known

- - - - Stimulants
        - Amphetamines (e.g., dexamfetamine, lisdexamfetamine, methylphenidate)

Please provide type and dose if known

- - - - Other medication
        - Please provide type and dose if known
    - Other, please specify
- Has the patient been offered any starting support prior to their regular treatment commencing?
  - Psychoeducation / recommended reading
  - Access to psychoeducational or pre-treatment groups
  - Other, please specify

**Clinician/Service reported information at start of treatment:**

- Eating disorder diagnosis – codes summarised elsewhere, need an option for no ED and an option for ‘eating disorder with no specific diagnosis given’
- Date of first treatment session
- Is the patient being seen through FREED (First Episode Rapid Early Intervention for Eating Disorders)?
  - No
  - Yes
- Physical health information – summarised elsewhere (height/weight/BMI/blood tests)
- Treatment plan using drop-down options, same as at assessment
- Any starting support provided prior to regular treatment commencing
  - Psychoeducation / recommended reading
  - Access to psychoeducational or pre-treatment groups
  - Other, please specify
- Therapist years of experience working with eating disorders
- Therapist professional group

**Clinician/Service reported information at end of treatment:**

- Eating disorder diagnosis – codes summarised elsewhere, need an option for no ED and an option for ‘eating disorder with no specific diagnosis given’
- Date of last treatment session
- Was treatment completed?
  - Yes
  - No, mutual decision to end early
    - As the patient moved away
    - Jointly agreed it wasn’t the right time for treatment
    - Referred to another service
    - Other reason (specify)
  - No, patient discontinued early
    - Reason if available
  - No, service decided to discontinue early
    - Reason if available
- Physical health information – summarised elsewhere (height/weight/BMI/blood tests)
- Treatment provided, multiple options able to be selected:
  - Inpatient
    - Specialist EDU inpatient treatment
      - Please enter admission and discharge dates
    - General medical ward inpatient treatment
      - Please enter admission and discharge dates
    - General psychiatric ward inpatient treatment
      - Please enter admission and discharge dates
  - Were the following provided as part of inpatient care?
    - Psychological therapy
      - To then provide the same response options as under outpatient psychological therapy (therapy models, if group/individual, and number of sessions)
    - Dietetics
    - Occupational therapy
    - Family therapy (not specifically focused on the eating disorder)
    - Carer support sessions or carer skills workshops
    - Peer support
    - Psychiatry or medical reviews
    - Nutritional supplements or prescriptions (please only select medications that were recommended or prescribed as part of eating disorder team input)
      - Multivitamin
      - Potassium supplements (e.g., Sando-K)
      - Magnesium supplements
      - Phosphate supplements
      - Calcium supplements
      - Iron/ferritin supplements
      - Vitamin D supplements
      - Other (please specify)
    - Psychotropic medication (please only select medications that were recommended or prescribed as part of eating disorder team input)
      - Antidepressants
        - SSRI antidepressants (e.g., citalopram, escitalopram, fluoxetine, paroxetine, sertraline)

Please provide type and dose if known

- - - - - SNRI antidepressants (e.g., atomoxetine, duloxetine, venlafaxine)

Please provide type and dose if known

- - - - - NASSA antidepressants (e.g., mirtazapine)

Please provide type and dose if known

- - - - - Tricyclic antidepressants (e.g., amitriptyline)

Please provide type and dose if known

- - - - - Other antidepressant

Please provide type and dose if known

- - - - Antipsychotic / atypical antipsychotic (e.g., olanzapine)
        - Please provide type and dose if known
      - Anxiolytics / anti-anxiety / insomnia / sedative medications
        - Anaesthetics (e.g., ketamine, propofol)

Please provide type and dose if known

- - - - - Antihistamines (e.g., diphenhydramine, promethazine)

Please provide type and dose if known

- - - - - Barbiturates (e.g., amobarbital, butalbital)

Please provide type and dose if known

- - - - - Benzodiazepines (e.g., diazepam, lorazepam, temazepam)

Please provide type and dose if known

- - - - - Buspirone

Please provide type and dose if known

- - - - - Melatonin

Please provide type and dose if known

- - - - - Muscle relaxants (e.g., clonidine, pregabalin)

Please provide type and dose if known

- - - - - Non-benzodiazepine hypnotics (e.g., zolpidem, zopiclone)

Please provide type and dose if known

- - - - - Other anti-anxiety or sedative medications

Please provide type and dose if known

- - - - Mood stabilisers (non antipsychotic)
        - Anticonvulsants (e.g. carbamazepine, lamotrigine)

Please provide type and dose if known

- - - - - Lithium

Please provide type and dose if known

- - - - Stimulants
        - Amphetamines (e.g., dexamfetamine, lisdexamfetamine, methylphenidate)

Please provide type and dose if known

- - - - Other medication
        - Please provide type and dose if known
    - Nursing reviews
    - Meal support
    - Other, please specify
  - Were the following used as part of inpatient care?
    - PEACE pathway adaptations for autism
      - No
      - Yes
    - Mental health act (involuntary treatment)
      - No
      - Yes – if yes, please specify section type
    - Nasogastric tube feeding
      - Not used
      - Used <5 days throughout the admission
      - Used >=5 days throughout the admission
    - Use of restraint
      - Not used
      - Used <5 times throughout the admission
      - Used >=5 times throughout the admission
  - Day-services
    - Full-time EDU day-services programme (9-5 Mon-Fri)
      - Please enter admission and discharge dates
    - Part-time EDU day-services programme (less than 9-5 Mon-Fri)
      - Please enter admission and discharge dates
    - Non eating disorder focused day-services care
      - Please enter admission and discharge dates
  - Were the following provided as part of day-services care?
    - Psychological therapy
      - To then provide the same response options as under outpatient psychological therapy (therapy models, if group/individual, and number of sessions)
    - Dietetics
    - Occupational therapy
    - Family therapy (not specifically focused on the eating disorder)
    - Carer support sessions or carer skills workshops
    - Peer support
    - Psychiatry or medical reviews
    - Nutritional supplements or prescriptions (please only select medications that were recommended or prescribed as part of eating disorder team input)
      - Same options as under inpatient care
    - Psychotropic medication (please only select medications that were recommended or prescribed as part of eating disorder team input)
      - Same options as under inpatient care
    - Nursing reviews
    - Meal support
    - Other, please specify
  - Were the following used as part of day-services care?
    - PEACE pathway adaptations for autism
      - No
      - Yes
    - Mental health act (e.g., community treatment order)
      - No
      - Yes – if yes, please provide details
    - Nasogastric tube feeding
      - Not used
      - Used <5 days throughout the admission
      - Used >=5 days throughout the admission
    - Use of restraint
      - Not used
      - Used <5 times throughout the admission
      - Used >=5 times throughout the admission
  - Intensive outreach, enhanced treatment team or home treatment team treatment
    - Eating disorder focused intensive outreach treatment
      - Please enter admission and discharge dates
    - Non eating disorder focused intensive outreach treatment
      - Please enter admission and discharge dates
  - Were the following provided as part of intensive outreach care?
    - Psychological therapy
      - To then provide the same response options as under outpatient psychological therapy (therapy models, if group/individual, and number of sessions)
    - Dietetics
    - Occupational therapy
    - Family therapy (not specifically focused on the eating disorder)
    - Carer support sessions or carer skills workshops
    - Peer support
    - Psychiatry or medical reviews
    - Nutritional supplements or prescriptions (please only select medications that were recommended or prescribed as part of eating disorder team input)
      - Same options as under inpatient care
    - Psychotropic medication (please only select medications that were recommended or prescribed as part of eating disorder team input)
      - Same options as under inpatient care
    - Nursing reviews
    - Meal support
    - Other, please specify
  - Were the following used as part of intensive outreach care?
    - PEACE pathway adaptations for autism
      - No
      - Yes
    - Mental health act (e.g., community treatment order)
      - No
      - Yes – if yes, please provide details
    - Nasogastric tube feeding
      - Not used
      - Used <5 days throughout the admission
      - Used >=5 days throughout the admission
    - Use of restraint
      - Not used
      - Used <5 times throughout the admission
      - Used >=5 times throughout the admission
  - Outpatient psychological therapy:
    - Guided self-help based on CBT
    - CBT-T (10-session CBT for eating disorders)
    - CBT-ED/CBT-E (CBT for eating disorders / Enhanced CBT for eating disorders
    - MANTRA (Maudsley model Anorexia Nervosa Treatment for Adults)
    - SSCM (Specialist Supportive Clinical Management)
    - FT-AN / FT-BN / FBT (Family based treatment for eating disorders)
    - Other
      - Focal psychodynamic psychotherapy
      - Cognitive analytical therapy
      - Schema therapy
      - Dialectical behaviour therapy
      - Radically open dialectical behaviour therapy
      - Other, please specify
  - After selecting outpatient therapy, to select if individual or group or a mix of individual + group
  - After selecting outpatient therapy and group/individual;
    - - How many therapy sessions were attended?
      - How many therapy sessions were offered but cancelled or not attended (DNA)?
  - Other outpatient support
    - Dietetics
    - Occupational therapy
    - Family therapy (not specifically focused on the eating disorder)
    - Carer support sessions or carer skills workshops
    - Peer support
    - Psychiatry or medical reviews
    - Nutritional supplements or prescriptions (please only select medications that were recommended or prescribed as part of eating disorder team input)
      - Same options as under inpatient care
    - Psychotropic medication (please only select medications that were recommended or prescribed as part of eating disorder team input)
      - Same options as under inpatient care
    - Nursing or physical health monitoring reviews
    - Other, please specify
  - Were the following used as part of outpatient treatment?
    - PEACE pathway adaptations for autism
      - No
      - Yes
    - Mental health act (e.g., community treatment order)
      - No
      - Yes – if yes, please provide details
  - Did the patient attend A&E during this episode of eating disorder treatment (whether directed by your team or otherwise)?
    - No
    - Yes, <5 times
    - Yes, >=5 times
  - Was the patient referred for physical health investigations or treatment by your service?
    - No
    - Yes, please specify
  - If transitions occurred between outpatient/day-patient/intensive outreach/inpatient treatment, would you describe treatment as integrated? I.e., were transitions across pathways planned, with no gap in care provision and with common treatment goals?
    - Yes
      - Was this guided by integrated CBT-E (I-CBTE) or another approach?
        - I-CBTE
        - Another approach

Please specify

- - - No
  - If a transition occurred from child/adolescent to adult eating disorder treatment, how was this transition managed? - Please select all that apply
    - There was a planned transition from the child/adolescent to adult team with advance meetings and no gap in care
    - There was a planned transition from the child/adolescent to adult team but with no advance meetings between the teams
    - There was a gap in treatment provision between the child/adolescent and adult teams
    - The transition between teams was not planned
    - The child/adolescent team continued to work with the young person beyond their 18^th^ birthday to support a smooth transition
    - The adult team started to work with the young person before their 18^th^ birthday to support a smooth transition
